# Improving Wearable PPG Monitoring with Morphology-Aware and Learning-Driven Heartbeat Detection †

**DOI:** 10.3390/s26216912

**Published:** 2026-10-31

**Authors:** Pietro Chierico, Daniel Suárez, Lorenzo Fácila, Fernando Hornero, Raúl Alcaraz, José J. Rieta

**Affiliations:** 1 BioMIT.org, Electronic Engineering Department, Universitat Politècnica de València, 46022 Valencia, Spain; pchierico@upv.es; 2 Benejúzar Health Center, Orihuela Health Department, 03390 Benejúzar, Spain; suarez_dan@gva.es; 3 Cardiology Department, General University Hospital Consortium of Valencia, 46014 Valencia, Spain; lfacila@gmail.com; 4 Cardiovascular Surgery Department, Hospital Clínico Universitario de Valencia, 46010 Valencia, Spain; hornero_fer@gva.es; 5 Research Group in Electronic, Biomedical and Telecommunication Engineering, University of Castilla-La Mancha, 16071 Cuenca, Spain; raul.alcaraz@uclm.es

**Keywords:** photoplethysmography, PPG, peak detection, heartbeat detection, template matching, synthetic noise, wearable sensors

## Abstract

Photoplethysmography (PPG) is a widely used, non-invasive technique for heart rate monitoring, but its reliability is often compromised by noise and motion artifacts (MAs). This study presents the Peakwise Correlation Pulse Detector (PCPD), an algorithm for detecting heartbeats directly from PPG signals without requiring electrocardiogram (ECG) signals during operation, evaluated here under clean and controlled synthetic pulse-free-noise conditions. PCPD follows a five-step pipeline: (1) signal segmentation with overlap, (2) sliding cross-correlation using high-quality heartbeat templates, (3) computation of the Minimum Correlation Curve (MCC), (4) feature extraction with machine learning-based classification, and (5) peak translation with constraint analysis. The method was evaluated on a clean dataset, comprising a newly collected PPG dataset with manually annotated signal quality and the publicly available BIDMC dataset, and on a noisy dataset that augments the clean data with synthetic pulse-free segments generated from band-limited Gaussian noise. This evaluation framework tests heartbeat detectors under ideal conditions and under a controlled non-informative-noise protocol. On clean signals, PCPD achieved balanced precision–recall scores of 99.13% on the proposed dataset (internal performance) and 97.04% on the independent BIDMC dataset. Under added synthetic pulse-free noise, PCPD achieved accuracies of 99.30% and 97.16% on the proposed and BIDMC datasets, respectively. In comparative experiments against eleven benchmark heartbeat detection algorithms, PCPD achieved the best performance among the evaluated implementations under the tested synthetic-noise protocol. These results support PCPD as a promising PPG-only peak detector and motivate further validation under real motion artifacts and for downstream signal quality assessment.

## 1. Introduction

Photoplethysmography (PPG) is a non-invasive and widely adopted technique commonly utilized in wearable devices to monitor a range of physiological signals, with a particular focus on indicators of cardiovascular health [1]. Its waveforms consist of repetitive cycles, each characterized by a primary systolic peak, corresponding to the pulse peak and indicative of systolic pressure [2]. This is typically succeeded by a lower-amplitude diastolic peak, representing diastolic pressure. The accuracy and reliability of PPG data, despite its widespread application, can be significantly influenced by various external factors, particularly motion artifacts (MAs) [3,4]. These artifacts, arising from daily activities of the subject under study, distort the PPG waveform, thus complicating analysis [5]. This issue becomes even more pronounced in conditions of low perfusion, where weak pulse waves and minimal differences between systolic and diastolic pressures result in physiological signals being easily masked by noise [6]. Studies have shown that MAs contribute significantly to erroneous measurements, accounting for 70–71% of false-positive pulse oximeter alarms [7,8]; this issue is particularly pronounced in apnea monitoring, where false desaturation alarms can reach rates as high as 85% [9]. Additionally, factors such as variations in skin tone, sensor placement, and ambient light conditions intensify signal degradation, undermining the accuracy of later automated signal analysis stages and subsequent physiological interpretation [10,11]. These challenges underscore the necessity of advanced signal processing techniques to mitigate motion artifacts and improve the dependability of PPG-based monitoring systems. Moreover, detecting heartbeats without relying on additional data such as electrocardiography (ECG) during operation can facilitate the standalone functionality of wearable devices [12]. This practical advantage does not remove the value of an independent physiological reference when validating detection performance. This requirement becomes particularly significant in applications where only smartphone-based PPG beat detection is required, emphasizing the need for standalone operation [13]. To unlock the full potential of PPG as a non-invasive and cost-effective monitoring tool, it is vital to develop advanced techniques that identify systolic peaks accurately using only PPG signals.

In the literature, various solutions have been proposed to address challenges in PPG-based beat detection. A significant number of these approaches rely on combining the PPG signal with additional synchronized data sources, such as ECG or accelerometer data, to improve detection accuracy [14]. However, such methods depend on the availability and precise synchronization of these auxiliary signals. A comprehensive benchmarking study [14] evaluated several peak detection algorithms, each employing distinct methodologies suited to different applications. These algorithms range from advanced techniques, such as scalogram analysis, to threshold-based methods optimized for specific signal types, such as intracranial pressure (ICP) [15,16,17]. Some algorithms performed particularly well in high-quality datasets, while others were more effective in datasets with PPG signals captured during physical activities [18]. This reliance on ECG as the reference in the validation process introduces a methodological limitation. Specifically, peaks labeled as true positives (TPs) by the ECG may not always be discernible in the PPG signal. Consequently, a beat detection algorithm that does not recognize these peaks is penalized, even though the absence of detection may be due to inherent differences in the physiological signals rather than algorithmic shortcomings. This discrepancy highlights the need for more robust and context-aware validation methodologies that account for the unique characteristics and limitations of PPG-based beat detection.

In contrast, this paper introduces the Peakwise Correlation Pulse Detector (PCPD) for heartbeat detection in PPG signals, emphasizing its resilience to controlled pulse-free noise and its ability to operate without auxiliary ECG or accelerometer signals during deployment. Performance is evaluated against manually verified PPG pulse peaks rather than an independent ECG reference. By identifying intrinsic PPG waveform patterns, PCPD separates plausible heartbeat signatures from non-informative noise maxima in the tested protocol. This establishes a basis for PPG-only peak detection and motivates further validation under real motion-artifact conditions.

This article extends the preliminary work reported in [19], which introduced the PCPD method, its five processing steps and a summary of its performance. Relative to that work, the present article adds: (i) the full mathematical formulation of the sliding correlation, the MCC and the classifier features; (ii) the annotation protocol, with inter-rater agreement, and the peak-level ground truth; (iii) the analysis of the number of templates; (iv) separate evaluations of clean signals (precision, recall, F1) and noisy signals (full confusion matrix); (v) the classifier selection and validation protocol; (vi) precision, recall and specificity for all benchmark methods and the per-segment variability of PCPD; (vii) an expanded discussion and the study limitations; (viii) processing time, an ablation study, a sensitivity analysis and an exploratory segment-level paired comparison; and (ix) a subject-independent evaluation on held-out subjects and with subject-wise cross-validation. Material carried over from the conference paper accounts for less than 20% of the present text. Corrections to figures and descriptions given in the conference paper are listed in Section 5.3.

This paper is structured as follows: Section 2 describes the datasets used for evaluating PCPD, including dataset creation, synthetic noise simulation, segment characteristics, and the beat detection evaluation methodology. Section 3 describes the PCPD algorithm, which consists of a five-step process, together with the validation strategy adopted. Section 4 presents the results on the evaluated datasets. Section 5 discusses these findings, compares PCPD with eleven benchmark methods and reports a subject-independent evaluation. Finally, Section 6 summarizes the main conclusions.

## 2. Materials

The evaluation was conducted using two dataset configurations: a clean dataset and a noisy dataset. The clean dataset includes a newly collected PPG dataset, specifically developed for this study, containing multi-subject recordings labeled for signal quality, and the publicly available BIDMC [20] database, widely used in physiological signal analysis. The noisy dataset was constructed by augmenting both components of the clean dataset with manually inserted pulse-free sub-segments. These segments consist of non-physiological noise and are designed to simulate controlled non-informative intervals such as temporary sensor disconnection. This setup enables assessment of the ability of PCPD to discriminate genuine physiological peaks from synthetic noise-induced artifacts under varying signal quality conditions.

### 2.1. Clean Dataset Description

The clean configuration comprises two datasets used to assess the performance of PCPD under high-quality conditions. The first dataset was specifically created for this research and comprises PPG signals collected from multiple subjects using a Polar Verity Sense device. The second dataset, BIDMC, was employed as a reference for comparison, as it includes physiological signals from critically ill patients recorded at the Beth Israel Deaconess Medical Center (BIDMC). The combination of these datasets provides a comprehensive framework for evaluating PCPD.

**Figure 1:**
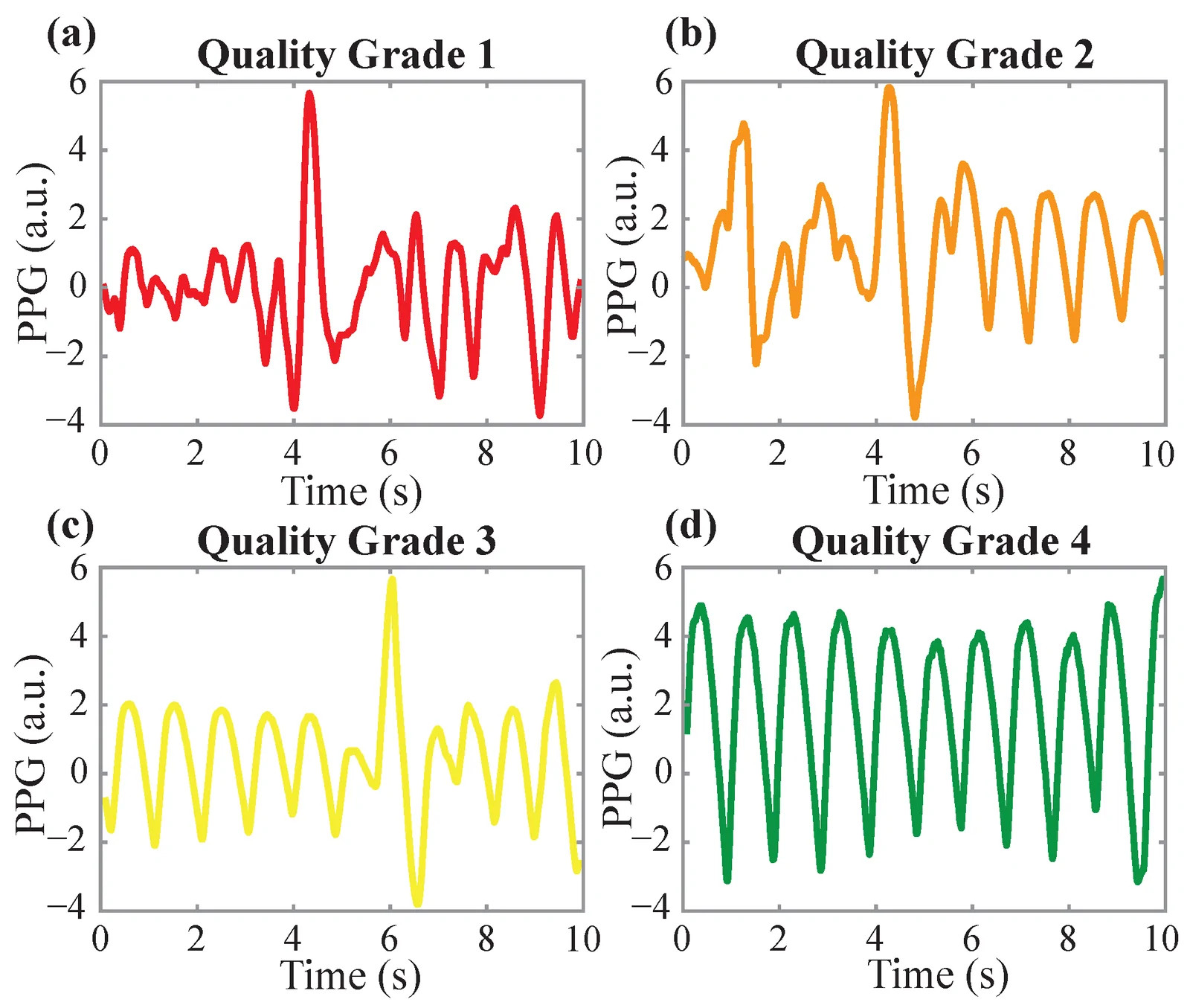
Four examples of PPG signal segments representing different signal-quality levels. (**a**) Grade 1: severely distorted signal with no reliably recognizable PPG pulse morphology. (**b**) Grade 2: poor-quality signal, with recognizable PPG pulses in less than 50% of the segment. (**c**) Grade 3: moderate-quality signal, with recognizable PPG pulses in more than 50% of the segment, although substantial artifacts remain. (**d**) Grade 4: high-quality signal with consistently recognizable PPG pulse morphology throughout the segment.

Due to the scarcity of publicly available datasets labeled with different quality levels in the literature [21,22], a novel dataset was developed to facilitate performance evaluation under realistic conditions. This dataset was obtained using a Polar Verity Sense device (Polar Electro Oy, Professorintie 5, 90440 Kempele, Finland), which is equipped with a three-channel optical heart rate (OHR) sensor operating at a sampling frequency of 55 Hz. The device outputs the raw, unprocessed PPG signal acquired by each of its three optical channels, without any internal filtering. The three channels were combined into a single PPG signal by averaging, following guidance reported by developers in discussions on the device’s official software development kit (SDK) repository (see discussion at https://github.com/polarofficial/polar-ble-sdk/issues/202 (accessed on 23 October 2026)), as averaging was found to reduce channel-specific noise relative to using a single channel alone. In total, 24 h of PPG signal data were collected from multiple subjects. To improve the accuracy of signal quality assessment and labeling, the dataset was segmented into 10-s windows. This segmentation approach was chosen to address the challenges of assigning a single quality label to longer windows, such as 60 s, where fluctuations in signal quality can be significant [23]. By using shorter segments, variations in signal quality could be captured with greater precision, resulting in a more accurate classification process. The dataset comprises 8640 segments, each manually labeled based on signal quality following the annotation protocol described below. The classification, shown in Figure 1, was performed using a four-tier grading system: Grade 1 corresponds to segments with no reliably recognizable PPG pulse morphology, Grade 2 includes segments where recognizable PPG pulses are present in less than half of the segment, Grade 3 indicates segments where recognizable PPG pulses are present in more than half of the segment, and Grade 4 represents segments with consistently recognizable PPG pulse morphology throughout the entire duration.

In this study, a pulse was considered recognizable when a clear, unambiguous systolic peak could be identified in the segment. The presence of secondary morphological features, such as the dicrotic notch, was not required for a pulse to be classified as recognizable, as such features are frequently attenuated or absent in real-world reflectance-mode PPG recordings from wrist-worn wearable sensors, unlike in clinical-grade contact PPG.

The 8640 segments were labeled independently by two annotators, both PhD students with prior experience in PPG signal analysis. Annotators were blinded to subject identity but not to the source recording device. Segments for which the two labels disagreed were subsequently reviewed and adjudicated by a supervising professor with expertise in PPG signal processing, whose judgment determined the final consensus grade; this consensus label constitutes the definitive ground truth used in this study. Inter-rater agreement between the two independent annotators was Cohen’s kappa = 0.47 (unweighted), corresponding to moderate agreement, and quadratic-weighted kappa = 0.76, corresponding to substantial agreement; the higher weighted value reflects that most disagreements occurred between adjacent quality grades on this ordinal four-tier scale, consistent with the graded nature of the classification task.

In addition to signal data, the dataset contains information from 50 subjects, with an average age of 54 years and varying health conditions, including cardiovascular diseases. Among the subjects, 80% have hypertension, and the average body mass index (BMI) is 28.1, with 60% classified as overweight or obese. The sample includes 60% women and 40% men, and 50% of the subjects engage in regular physical activity. The mean heart rate is 77 beats per minute (bpm). Table 1 reports the resulting distribution of quality grades across the 24-h dataset.

For comparative analysis, the BIDMC [20] dataset was utilized. This dataset originates from the MIMIC II [24] Matched Waveform Database and comprises physiological signals and numerical parameters collected from critically ill patients during hospitalization at Beth Israel Deaconess Medical Center in Boston, USA. The dataset contains 53 recordings, each lasting eight minutes, and includes various physiological signals such as PPG, impedance respiratory signals, and electrocardiograms, all sampled at 125 Hz. The BIDMC dataset has been widely used for evaluating algorithms designed to estimate respiratory rate from PPG signals. Incorporating this dataset into our study provides an evaluation on independent, publicly available data.

### 2.2. Noisy Dataset Description

To evaluate the specificity of PCPD in distinguishing true PPG morphology from noise-induced fluctuations, a controlled dataset incorporating pulse-free sub-segments was developed. This experimental design enables the assessment of the algorithm’s capability to accurately identify physiological peaks while rejecting maxima generated by the synthetic pulse-free noise. This controlled protocol isolates rejection of clearly non-physiological candidates; it does not reproduce the morphology or temporal structure of real motion artifacts and does not by itself validate PCPD as a signal quality assessment method.

For this evaluation, the clean dataset served as the foundation for assessing the impact of synthetic pulse-free noise on a method designed to identify pulse-related features. Each dataset was divided into 10-s segments, yielding 4462 segments for the proposed dataset and 2544 segments for the BIDMC dataset.

To introduce noise into the dataset in a controlled manner, a manual noise injection process was implemented. By adding synthetic pulse-free noise, we simulated a condition where the PPG signal contains no recognizable pulse patterns, providing an opportunity to test the ability of the system to distinguish true heartbeats from non-informative maxima [25]. Using synthetic noise instead of noise derived from low-quality PPG signals avoids the risk of introducing ambiguous waveform morphology into the ground truth. If the noise were based on low-quality PPG signals, artifacts might resemble true heartbeats and make the labels ambiguous. The primary objective of this approach was to evaluate the beat detection method under partial pulse-free noise conditions. By introducing noise in short windows within each clean PPG segment, we aimed to simulate controlled transitions between informative and non-informative signal intervals, such as partial sensor detachment. This protocol allows assessment of the ability of the method to detect true peaks while rejecting noise-induced maxima, but it should not be interpreted as a full validation under real motion artifacts. Because the injected noise is deliberately unambiguous and lacks pulse-like structure, this experiment should be regarded as a controlled specificity test rather than as a more demanding or lower-bound surrogate for real-world motion artifacts. The extent to which the performance reported here generalizes to real-world PPG artifacts therefore remains to be established through validation on datasets containing naturally occurring noise, which we identify as an important direction for future work.

**Figure 2:**
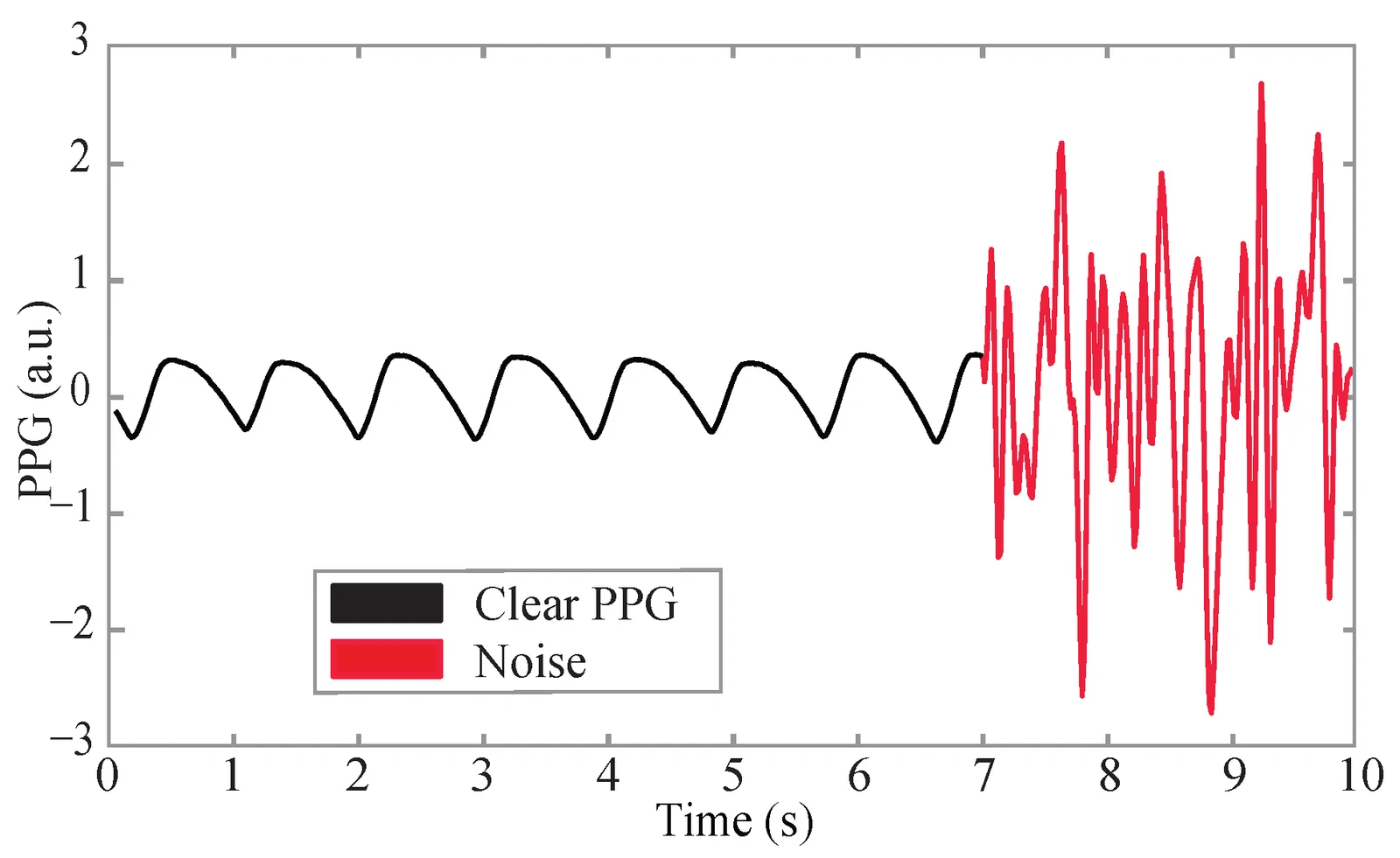
An example of a signal segment containing both a high-quality informative sub-segment and a manually added pulse-free noisy sub-segment (highlighted in red). The informative sub-segment is the raw, unfiltered PPG signal obtained by averaging the three optical channels of the Polar Verity Sense (Section 2.1); its smooth, near-sinusoidal morphology is typical of reflectance-mode PPG acquired at the wrist. The noisy section was introduced to simulate a non-informative signal, allowing for better evaluation of PCPD. (Adapted from our previous work [19]).

Noise, as depicted in Figure 2, was introduced into the dataset with varying segment lengths. The intervals of these segments were drawn from a Gaussian distribution with a mean of 2.5 s and a standard deviation of 0.75 s. Additionally, segment durations were constrained within a range of 0.5 to 4 s to ensure they remained within a reasonable time frame. The noise signal was generated from a normal distribution with zero mean and unit variance. To ensure that the noise amplitude was proportional to the variability of the original PPG signal, the generated noise was scaled to a standard deviation equal to the standard deviation of the replaced portion of the segment multiplied by an amplification factor of 10 for the proposed dataset, and by a factor between 2 and 10 increasing with the mean absolute amplitude of the clean portion for BIDMC. The scaled Gaussian noise was then band-limited to 0.5–8 Hz [26]. This range was chosen because frequencies outside this band are typically filtered out during preprocessing steps in PPG signal processing pipelines. Therefore, the resulting disturbance should be described as band-limited Gaussian noise. The Gaussian distribution and amplitude scaling were used to generate a controlled dataset; a single noise configuration was evaluated for each dataset.

A critical aspect of the dataset construction was maintaining balance in peak representation. Since noise segments inherently contain a higher density of spurious peaks, their duration was intentionally constrained to prevent overrepresentation of false peaks in the dataset. Had the noisy segments been as long as clean PPG segments, the dataset would have been skewed toward an excessive number of detected peaks in noise-dominated regions, potentially biasing the evaluation. By ensuring a balanced representation of true and false peaks, the ability of the beat detection method to distinguish physiological peaks from noise-induced artifacts can be accurately assessed.

The “Clear PPG” segment shown in Figure 2 corresponds to the raw, unfiltered signal obtained by averaging the three optical channels of the Polar Verity Sense, as described in Section 2.1. Its comparatively smooth, near-sinusoidal morphology reflects typical characteristics of reflectance-mode PPG acquired at the wrist in real-world wearable conditions, which generally exhibits a less pronounced systolic upstroke and dicrotic features than clinical transmission-mode PPG recorded at the finger.

### 2.3. Methodology for Beat Detection Evaluation

Accurately evaluating PCPD requires a validation framework in which noise can interfere with physiological signals while the PPG-based ground truth remains interpretable. Unlike previous approaches that relied on ECG as a reference [14], this study exclusively utilizes the PPG signal to assess detection performance. A key limitation of using ECG as a reference is that a peak considered valid in ECG may coincide with noise in the PPG signal. Consequently, if a beat detection algorithm fails to identify such a peak in PPG, it would be unfairly penalized, even though the peak is not actually valid in the PPG data. Accordingly, the ground truth adopted throughout this study represents detectable PPG pulses, i.e., waveform features manually confirmed as genuine systolic peaks in the PPG signal itself, rather than physiological cardiac events independently confirmed by a reference signal such as ECG. Ensuring that the evaluation provides an accurate measure of algorithmic performance in practical applications requires a methodology that differentiates between true positives (TPs), true negatives (TNs), false positives (FPs), and false negatives (FNs).

To identify TPs within clean signal segments, the findpeaks function from MATLAB (R2024a, The MathWorks, Inc., Natick, MA, USA) was employed with a minimum peak distance of 0.4 s, that is, $0.4f_{s}$ samples, where $f_{s}$ is the PPG sampling frequency. The 0.4-s minimum peak distance corresponds to an upper bound of 150 beats per minute (bpm), which is a conservative and realistic constraint given that the PPG recordings analyzed here were collected during typical daily activities, for which heart rates rarely exceed 150 bpm rather than the extreme-exertion values reflected in the broader 40–220 bpm physiological range considered elsewhere in this study. This threshold additionally prevents the dicrotic notch, a secondary waveform feature that can otherwise resemble a local maximum, from being erroneously detected as a separate peak. Moreover, since every candidate identified by findpeaks was subsequently manually inspected, with erroneous peaks removed and missed valid peaks added, any genuine peak that might have been suppressed by this distance constraint would have been recovered during manual verification. This manual inspection was performed through a dedicated graphical application. Unlike the signal-quality grading described in Section 2.1, which involved two independent annotators, this peak-level verification consisted of a manual quality-control check performed by a single reviewer, who corrected the output of the automated findpeaks-based candidate detection by removing spurious candidates and adding missed valid peaks. As this step was performed by a single reviewer, no inter-rater agreement statistic applies to the peak-level annotations. This process ensured that the final labeled dataset provided a reliable ground truth for algorithm assessment. For the identification of TNs within noisy segments, the findpeaks function was applied without parameter calibration, allowing for the detection of all local maxima in the signal. This approach ensured that any peak-like structures emerging due to noise, rather than genuine physiological events, were accurately identified.

Formally, this evaluation framework treats each local maximum returned by findpeaks as a peak candidate and formulates beat detection as a binary classification problem over this candidate set. A true positive (TP) is a candidate that corresponds to a genuine physiological PPG pulse and is correctly identified as such by the detection algorithm; a false negative (FN) is a genuine PPG pulse candidate that the algorithm fails to identify. A true negative (TN) is a candidate arising from non-physiological noise that is correctly rejected by the algorithm; a false positive (FP) is a noise-induced candidate that the algorithm incorrectly classifies as a valid peak.

Detected peaks were matched to reference peaks using an exact, one-to-one sample-index correspondence, without applying any temporal tolerance window; a detected peak was counted as a true positive only if it coincided exactly with the sample index of a reference peak.

The clean evaluation set includes 49,626 manually verified real peaks from the Grade-4 subset of the proposed Polar dataset (Section 2.1), and 37,035 manually verified real peaks from the BIDMC dataset. In the noisy evaluation, the candidate set comprised 134,643 local maxima in total. Of these candidates, 59,837 were valid pulse candidates (TPs + FNs) and 74,806 were invalid noise candidates (FPs + TNs), corresponding to 44.44% and 55.56% of the candidate set, respectively. This distribution provides a balanced representation of both valid and invalid candidates, enabling assessment of beat detection performance under controlled pulse-free noise conditions.

## 3. Methods

This section describes the methodological framework employed in the PCPD algorithm for peak detection in PPG signals. The algorithm consists of five sequential steps, each designed to enhance accuracy and reliability in identifying valid systolic peaks while mitigating the impact of controlled pulse-free noise and signal variability.

### 3.1. Signal Segmentation and Overlap

The first step of the algorithm involves segmenting the signal and overlapping it within 1-s windows, ensuring that relevant peaks are fully captured and reducing the risk of misclassification due to boundary effects. The choice of a 1-s overlap is justified by physiological considerations [27]. Specifically, this duration ensures that any peak near the edges of a segment is fully captured within the overlapping region, reducing the likelihood of misclassification due to incomplete data. Given the range of heart rates considered, spanning from 40 to 220 beats per minute (bpm), a 1-s overlap is sufficient to encompass the full waveform of any beat, including the most demanding case, that of the lowest heart rate considered [27,28]. Specifically, at 40 bpm the period of a full cardiac cycle is 1.5 s, so a beat whose peak lies close to the segment boundary starts at most half a cardiac cycle, that is 0.75 s, before that peak, and therefore falls within the overlapping region. This ensures that even in the case of the lowest heart rate, the peak is adequately captured within the window. As a result, the signal is divided into multiple segments, each of which is processed independently.

### 3.2. Sliding Correlation with Ideal Beats

**Figure 3:**
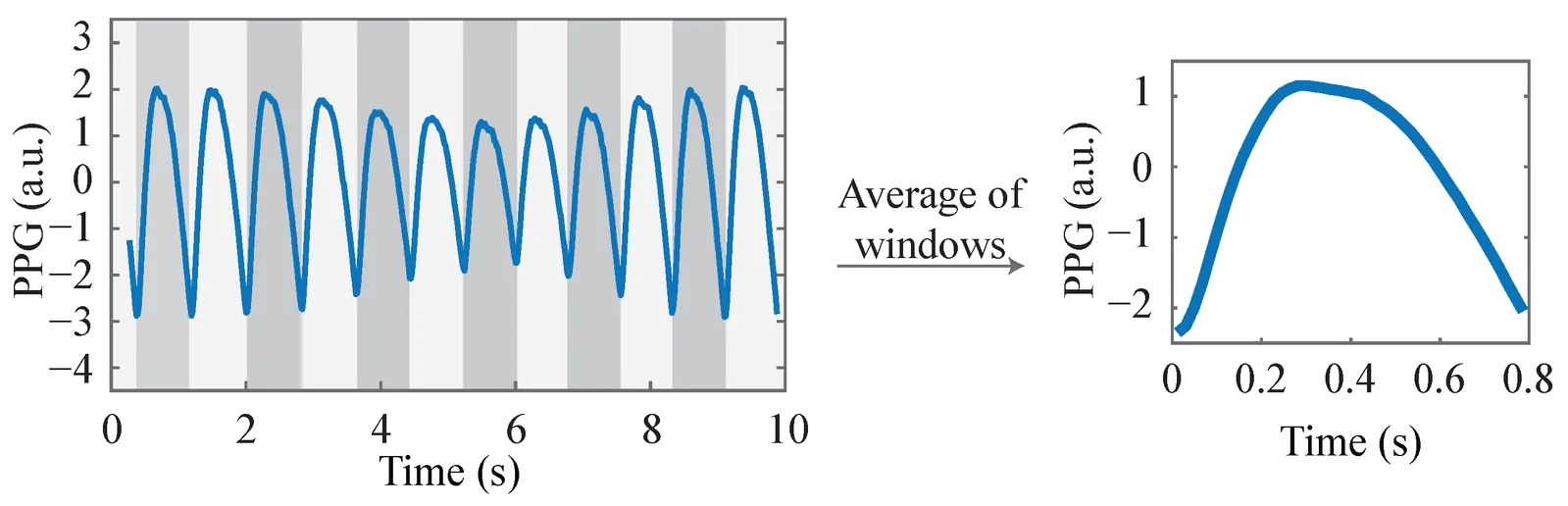
Visualization of high-quality signal segments used to generate ideal beat templates. The gray regions highlight individual beats, which are averaged to create robust high-quality reference beats.

The second step of PCPD involves performing cross-correlation between the segmented signal and a set of ideal beats. Each ideal window is obtained by averaging the beats of one high-quality (Grade-4) segment (the segments used are described in Section 3.4), thereby yielding robust template waveforms. The mean duration of these ideal beats was 854 ± 127 ms, corresponding to heart rates of 61–83 bpm. Although the subsequent classifier operates on shape-derived MCC features rather than directly on beat duration, the initial cross-correlation still uses templates drawn from this relatively narrow resting-rate range. Performance has not been analyzed separately across heart-rate strata; therefore, the 40–220 bpm plausibility constraint should not be interpreted as evidence of invariant detection performance throughout that entire range. A “window” or “beat” refers to the characteristic PPG curve, which is defined as the space between two minima, with a maximum value occurring between them, as explained in the introduction. As shown in Figure 3, the individual beats are highlighted by the shaded regions. Additionally, this figure clarifies, through an example, the concept of averaging all the beats from certain high-quality segments to create a high-quality window.

To quantify the similarity between the signal segment and the ideal beats, the Pearson correlation coefficient, denoted as *r*, is used. The Pearson correlation coefficient is defined as:

(1) $$r=\frac{\sum_{i=1}^{n}\left(X_{i}-\bar{X}\right)\left(Y_{i}-\bar{Y}\right)}{\sqrt{\sum_{i=1}^{n}{\left(X_{i}-\bar{X}\right)}^{2}}\sqrt{\sum_{i=1}^{n}{\left(Y_{i}-\bar{Y}\right)}^{2}}}$$

where $X_{i}$ and $Y_{i}$ represent the values of the segmented signal and the ideal beat at time index *i*, respectively. The terms $\bar{X}$ and $\bar{Y}$ denote the mean values of *X* and *Y*, and *n* is the length of the segment being analyzed.

For each signal segment, the ideal beats are systematically slid across the entire segment. Let $Y_{j,i}$ denote sample *i* of template *j*, with template length $n_{j}$, and let $X_{t+i}$ denote the corresponding samples of the PPG segment starting at position *t*. The template-specific sliding correlation is computed as

(2) $$r_{j}\left(t\right)=\frac{\sum_{i=1}^{n_{j}}\left(X_{t+i}-{\bar{X}}_{t,j}\right)\left(Y_{j,i}-{\bar{Y}}_{j}\right)}{\sqrt{\sum_{i=1}^{n_{j}}{\left(X_{t+i}-{\bar{X}}_{t,j}\right)}^{2}}\sqrt{\sum_{i=1}^{n_{j}}{\left(Y_{j,i}-{\bar{Y}}_{j}\right)}^{2}}},$$

where ${\bar{X}}_{t,j}$ is the mean of the segment samples covered by template *j* at position *t*, and ${\bar{Y}}_{j}$ is the mean of template *j*. Correlations are evaluated only at positions where the full template lies inside the segment; no zero padding is used at the segment boundaries. This calculation is repeated for each ideal beat, producing a set of correlation values for every valid time point within the segment.

**Figure 4:**
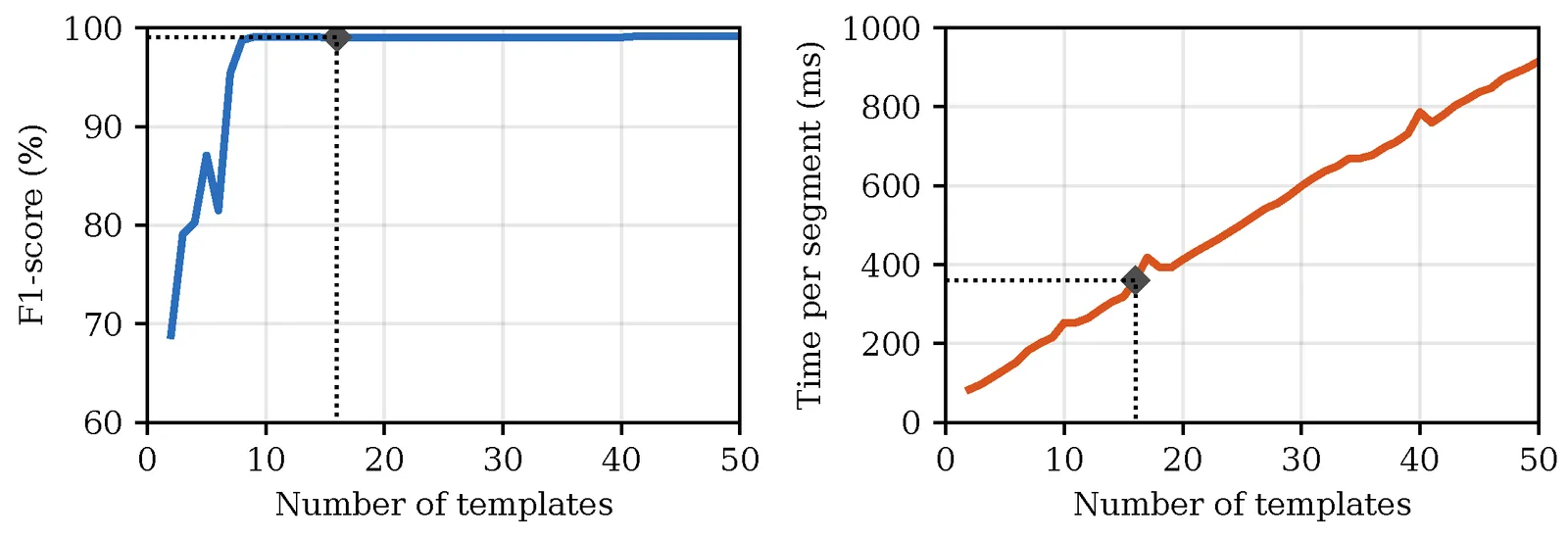
Evaluation of the window reduction process. The (**left**) graph shows the relationship between the number of windows used for cross-correlation and detection performance. The (**right**) graph shows the relationship between the computational time and the number of windows used. Both graphs illustrate the trade-off between computational efficiency and detection performance, showing that reducing the number of ideal beats significantly decreases execution time while preserving peak detection performance. Execution time is given per 10-s segment (median over the 100 segments; single thread, MATLAB R2023b, AMD Ryzen 5 5600G). The dotted lines and diamond markers indicate the selected configuration of 16 templates.

Initially, 50 ideal beats were used, obtained from the first 50 Grade-4 segments of the dataset. To optimize computational efficiency while maintaining accuracy, the number of ideal beats was progressively reduced to 16. This reduction significantly decreased execution time while preserving peak detection performance. To explore the impact of window reduction, a progressive approach was employed using high-quality templates, varying the number of windows from 2 to 50. The analysis was conducted on 100 randomly selected high-quality (Grade-4) segments, where PCPD was applied to detect peaks. The detected peaks were then compared against manually labeled reference peaks to assess detection performance. Graphical analysis, as presented in Figure 4, illustrates the relationship between execution time and the number of windows. An approximately linear increase was observed, indicating that reducing the number of windows improves computational efficiency. In terms of detection performance, results demonstrated a saturating trend, having reached its plateau at 16 windows. Because the template count was chosen on segments of the same dataset, this analysis supports an internal design decision rather than independent validation of the value 16.

### 3.3. Minimum Correlation Curve (MCC)

For each point in the PPG signal, 16 correlation curves are computed. The Minimum Correlation Curve (MCC) is then defined as

(3) $$\mathrm{MCC}\left(t\right)=\min_{j\in \{1,\ldots ,16\}}r_{j}\left(t\right).$$

**Figure 5:**
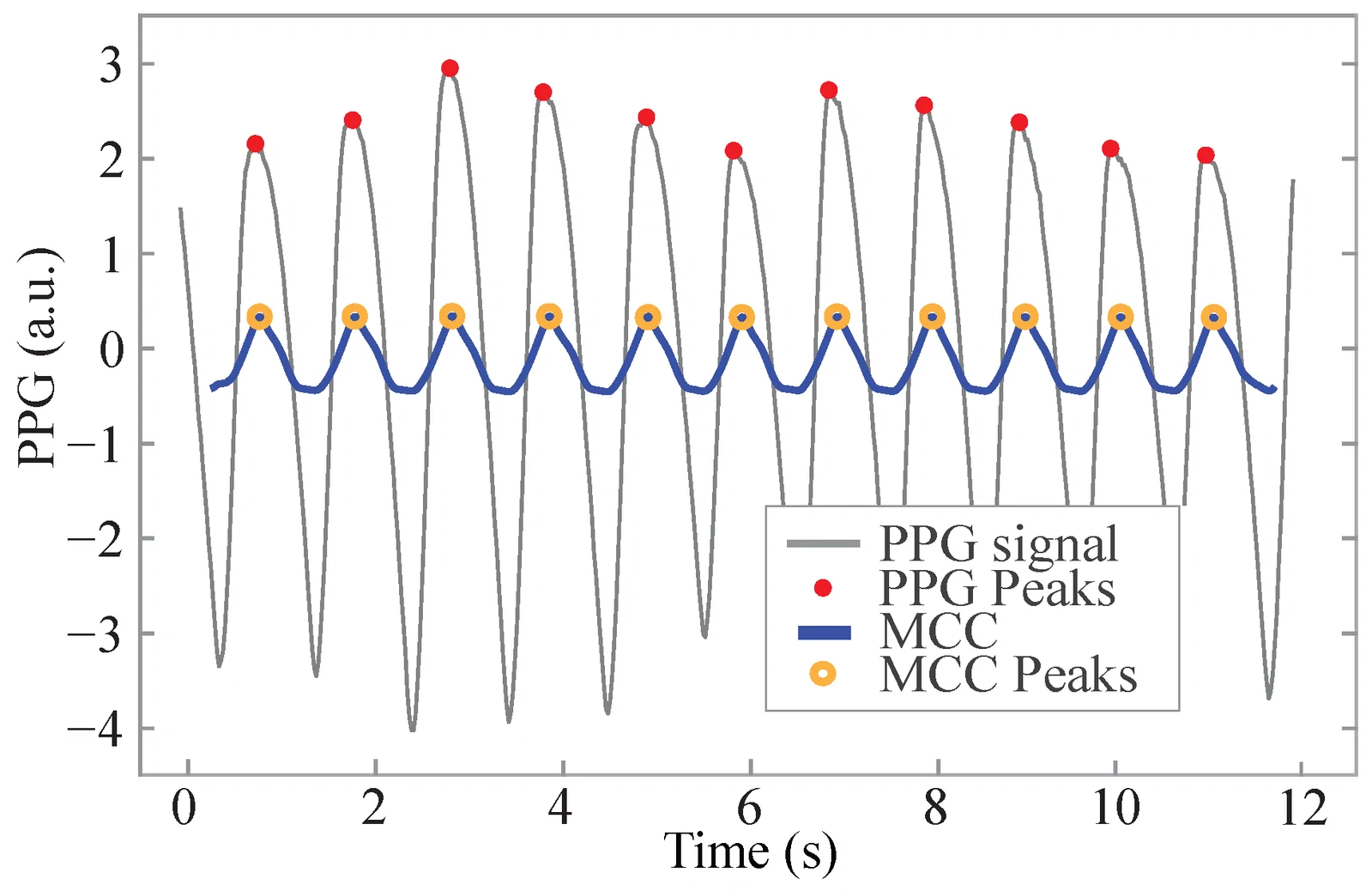
A segment of the PPG signal with the MCC overlaid, highlighting the matching peak timings. The MCC is constructed through cross-correlation of high-quality windows, meaning that when the MCC reaches a maximum, the templates are highly correlated with the corresponding PPG window.

The MCC can be regarded as a minimum-selection variant of multi-template cross-correlation; its contribution lies in using the least favorable template match as a strict criterion for peak validity. The advantage of the MCC lies in its geometric symmetry, which contrasts with the typically asymmetric morphology of the PPG signal. This symmetry is a direct result of using high-quality templates for correlation, ensuring that the idealized peak patterns are well-formed and consistent. Moreover, by selecting the minimum correlation value at each time point, the MCC offers a conservative measure of template agreement compared with maximum or average correlation values. The minimum correlation emphasizes the worst-case alignment at each point, focusing on the most challenging template match. This approach produces a sharper, more selective curve with a well-defined peak, making it easier to pinpoint the optimal alignment between the signal and the expected template. These characteristics, shown in Figure 5, make the MCC particularly effective for detecting and validating peaks with greater precision and reliability.

### 3.4. Binary Machine Learning Classification

Once the MCC is calculated, the next phase of the algorithm’s decision-making process begins. In this step, each candidate beat window is analyzed and submitted to a binary classifier. The model used for this classification task is a quadratic discriminant classifier. This model was selected after evaluating several other approaches, such as classification trees, support vector machines, and neural networks. This comparison formed part of the exploratory development process; because complete comparative results and a nested, subject-level model-selection protocol are not reported, it should not be interpreted as a formal demonstration of superiority over the other classifier families.

To train the classifier, labeled 10-s segments from the dataset described in Section 2.1 were used. Importantly, only signals with labels 1 and 4 were selected to prevent the model from learning segments with partially high and partially low quality, ensuring a more distinct separation between valid and invalid beats.

The classifier was trained on a balanced pool of 2002 labeled 10-s segments (Grade 1 and Grade 4 only, 50/50 class ratio), randomly split into a training set (80%) and a held-out test set (20%), with no duplicate segments between the two sets. Because this was a segment-level rather than a subject-stratified split, segments from the same subject may be present in both subsets. The internal classifier metrics may therefore be optimistic with respect to generalization to unseen subjects and should be interpreted as segment-level internal validation. On the held-out test set, the classifier achieved an accuracy of 97.25%, sensitivity of 97.10%, specificity of 97.41%, precision of 97.57%, F1-score of 97.34%, and an area under the ROC curve of 0.997 (confusion matrix: 201 true positives, 188 true negatives, 5 false positives, 6 false negatives), showing strong discrimination within this internal segment-level evaluation.

To train and apply the model, the following features are calculated for each candidate beat window of the MCC:

- **Skewness:** This statistical measure quantifies the asymmetry of a probability distribution relative to its mean. It is calculated based on the deviations of individual values $x_{i}$ from the mean $\bar{x}$, standardized by the standard deviation σ. The total number of observations, *N*, determines the scale of the summation. A positive skewness value indicates a distribution with a longer tail on the right, while a negative value indicates a longer tail on the left [29,30]. The formula for skewness is:
  (4) $$S=\frac{1}{N}\sum_{i=1}^{N}{\left(\frac{x_{i}-\bar{x}}{\sigma }\right)}^{3}$$
- **Ratio of Area (RoA):** This feature represents the ratio between the baseline-corrected area under the MCC window and the area of a rectangle constructed from the larger edge minimum and the maximum central value of the window. Let $b=\max(x_{L},x_{R})$, where $x_{L}$ and $x_{R}$ are the minimum values on the left and right sides of the window, respectively. The RoA is computed as
  (5) $$RoA=\frac{\sum_{k=1}^{w_{s}}\left(MCC_{k}-b\right)\Delta t}{(x_{C}-b)\cdot w},$$
  where $x_{C}$ is the maximum central value of the window, $w_{s}$ is the number of samples in the window, Δt is the sampling interval, and $w=w_{s}\Delta t$ is the window duration. Windows with $\left(x_{C}-b\right)$ close to zero are marked as invalid to avoid an unstable denominator.

**Figure 6:**
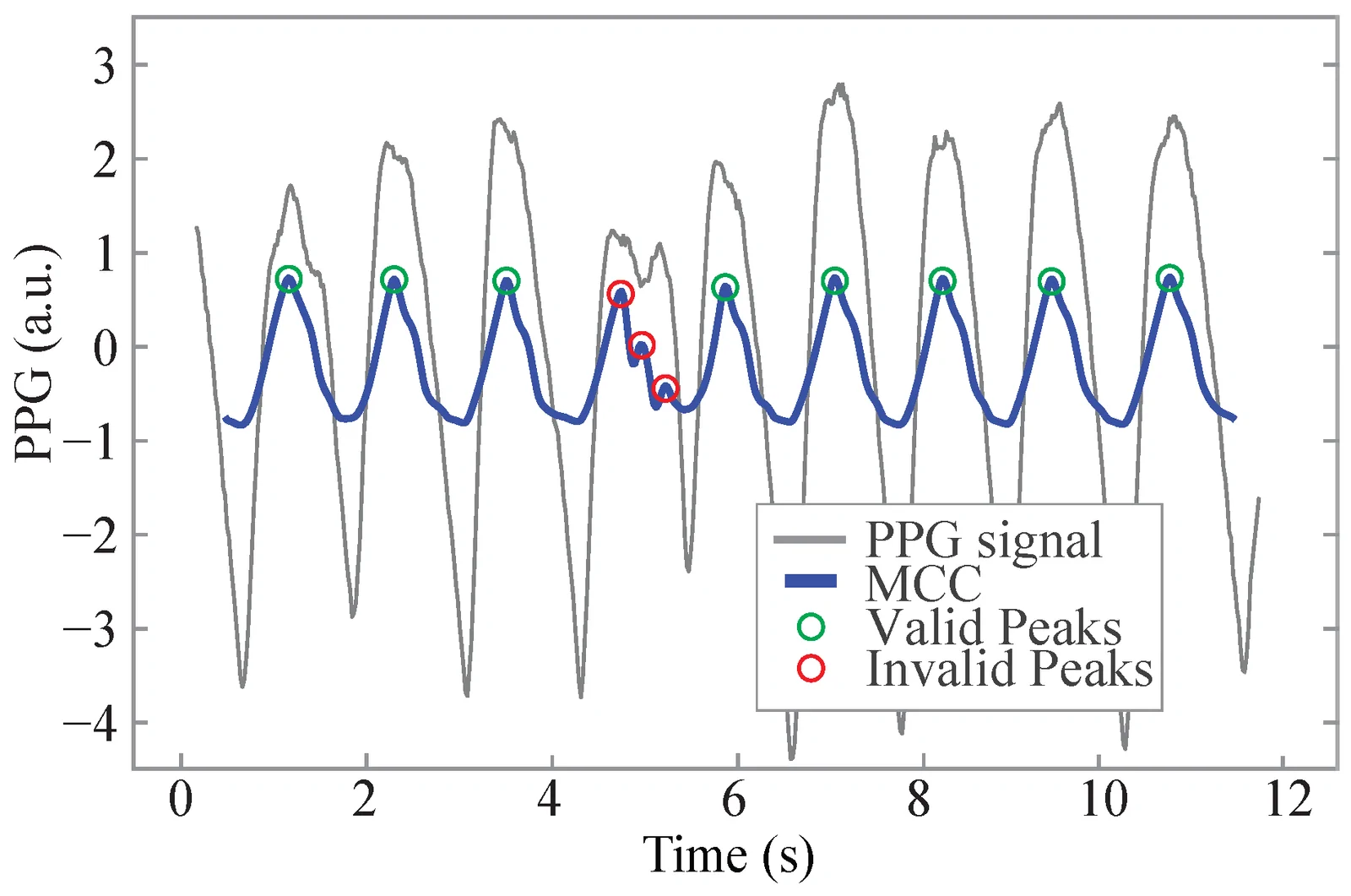
A segment of PPG with the MCC overlaid, illustrating the peak validation process. The quadratic discriminant classifier and constraint verification classify peaks into valid (green) and invalid (red) categories. (Adapted from our previous work [19]).

For each candidate window, skewness and RoA are used directly as a two-feature vector (S,RoA). During feature selection, multiple conventional statistical and signal-based features were tested, including standard deviation, mean, energy, and perfusion. However, morphological features demonstrated superior relevance in distinguishing valid from invalid candidate peaks. To maintain model simplicity and interpretability, skewness and RoA were selected as the final features. The trained model classifies each candidate window as valid or invalid based on this two-feature representation. Figure 6 shows an example of this classification stage applied to the MCC of a PPG segment. The classifier was trained on segment-level features (median RoA and median skewness over the candidate windows of each segment, labeled with the segment grade); the 400 test items above are therefore segments. At run time, the model is applied to the (S,RoA) pair of each candidate window.

Segments are assigned to subjects through the fixed block structure of the dataset (172 consecutive 10-s segments per subject, 212 for the last). The 16 templates are the mean beats of the first 16 Grade-4 segments in dataset order, all from a single subject; no other selection criterion was applied among the initial 50. The classifier pool comprised 1001 Grade-1 segments (subjects 1–21) and 1001 Grade-4 segments (subjects 1–12). The evaluation sets comprise all 4462 Grade-4 segments of the 50 subjects; hence 1001 of them (22.4%), from subjects 1–12, were also in the classifier pool, and subjects 22–50 (29 subjects, 3094 segments) were not used to build the templates or to train the classifier; segments from these subjects were, however, included in the template-count analysis of Figure 4. Subject-independent results are reported in Section 5.4.

The classifier is the same model as in the conference paper [19], which reported about seven hours of training data and a five-fold cross-validation accuracy of 97.2%. The classifier pool comprises 2002 segments (about 5.6 h), and the performance reported here is that of the single segment-level 80/20 hold-out described above.

### 3.5. Peak Translation and Constraint Analysis

Once candidate peaks are classified as valid or invalid, valid MCC peaks are matched to the original PPG peaks within a search window of ±10 samples centered on each MCC maximum. It is important to note that the peak of the correlation from the MCC will not always be perfectly aligned with the PPG peak. This misalignment arises due to various factors, including noise and the fact that each beat is distinct, meaning it is unlikely to match precisely with the “ideal” high-quality beats. Because the window is defined in samples, it corresponds to ±182 ms in the proposed dataset, sampled at 55 Hz, and to ±80 ms in BIDMC, sampled at 125 Hz. A wider window tolerates larger misalignments but increases the chance of including a neighboring beat at high heart rates; the value of ±10 samples was retained as a compromise, and its effect is examined in the sensitivity analysis (Section 5.6). After the classifier has labeled the candidates and the peaks have been translated, further analysis is conducted to ensure they meet additional constraints:

1. **Heart Rate (HR) Limits**: The peak is checked to confirm that the estimated heart rate falls within the operational limits used in this study, specifically between 40 and 220 bpm [27,28].
2. **Symmetry of Window Edges**: The left and right limbs of the window are compared. If the widths of these limbs differ by more than 50%, the peak is marked as invalid. This threshold-based control helps eliminate peaks that are morphologically correct but temporally misplaced.

**Figure 7:**
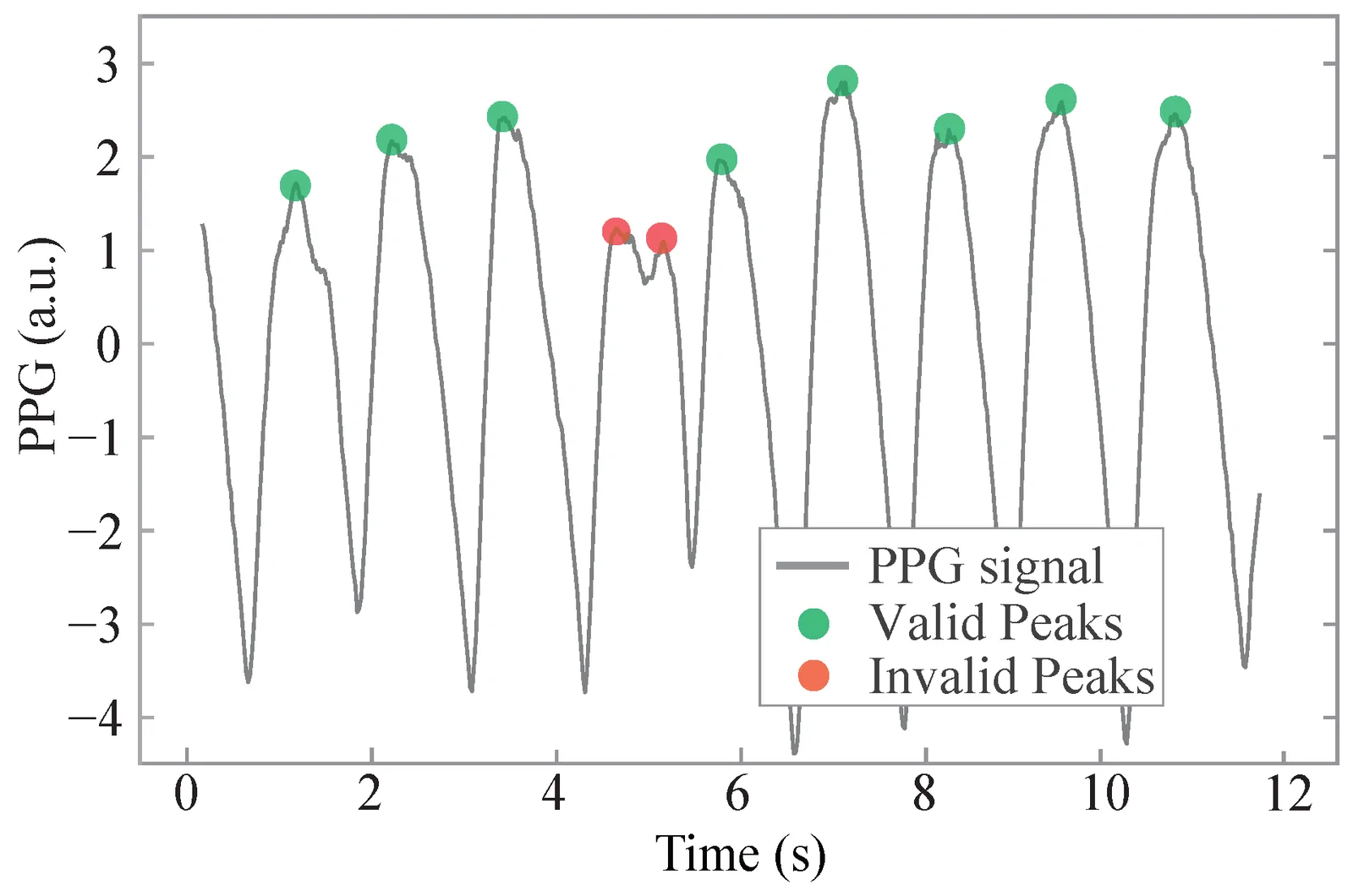
A segment of the PPG signal showing valid (green) and invalid (red) peaks at the end of the peak validation process. After validation using the MCC method, the peaks are mapped onto the PPG signal, providing a clear distinction between accepted and rejected peaks.

An example of the validated peaks can be seen in Figure 7, where valid (green) and invalid (red) peaks are mapped onto the PPG signal after the validation process.

### 3.6. Validation Strategy of the Method

To evaluate the PCPD algorithm, a comprehensive validation strategy was implemented. The goal was to assess the susceptibility of the method to controlled pulse-free noise and its overall performance in detecting significant peaks.

The evaluation was conducted using the two datasets previously described in Section 2.1: (i) a subset of high-quality signal segments extracted from 24-h recordings and (ii) the publicly available BIDMC dataset, which is a subset of MIMIC II. These datasets were tested under two different conditions. In the first condition, the datasets were analyzed without the addition of manual noise, using clean signals. In the second condition, synthetic band-limited Gaussian noise mimicking pulse-free intervals was added, as described in Section 2.2, to evaluate the specificity of the method, ensuring that it identifies PPG-derived peaks rather than detecting maxima in general. This allowed testing under high-quality signal conditions as a control and under controlled non-informative signal intervals as a stress test. Because the noisy condition is synthetic, it should be interpreted as a controlled pulse-free-noise evaluation rather than a complete validation under all real-world signal distortions.

Preprocessing differed between the two datasets and was identical for PCPD and the benchmark methods within each dataset. For the proposed dataset, the channel-averaged signal (Section 2.1) was used without any filtering in this study; the benchmark methods received the same signal and applied only their own internal preprocessing (Section 5.3). For BIDMC, the PPG was filtered with a fourth-order zero-phase Butterworth band-pass filter (0.5–8 Hz) before being processed by PCPD and by all benchmark methods; in the noisy condition, the synthetic noise was added to the filtered signal.

To quantify the performance of the PCPD pipeline, a detailed set of evaluation metrics was computed, following the definitions given in Section 2.3. For the noisy signal dataset, TPs, FNs, FPs, and TNs were computed, allowing calculation of standard accuracy, precision, recall, and F1 score. For the clean dataset, TNs do not exist, meaning only TPs, FPs, and FNs can be used. Consequently, precision, recall, and F1 score were computed for the clean dataset. The summary column reported for clean signals is therefore the mean of precision and recall, not standard accuracy; it is referred to as a balanced precision–recall score.

### 3.7. Overview of the PCPD Algorithm

**Figure 8:**
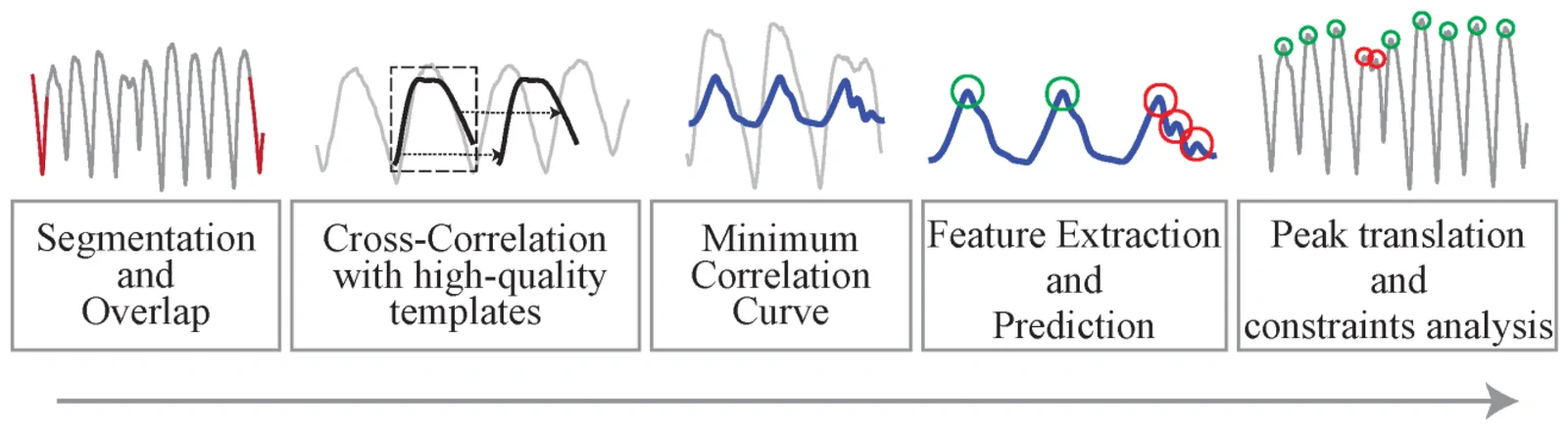
Overview of the PCPD algorithm pipeline (from left to right), which consists of five sequential steps: segmentation and overlap, cross-correlation with high-quality templates, MCC generation, feature extraction and prediction using machine learning, peak translation and constraints analysis. (Adapted from our previous work [19]).

To summarize, Figure 8 illustrates the five-step pipeline of the PCPD algorithm for peak detection in physiological signals. The process begins with segmentation and overlap, where the signal is divided into overlapping segments to prevent peak misclassification due to boundary effects. Next, cross-correlation with high-quality templates is performed, where each segment is compared against a set of ideal beats using Pearson correlation to assess waveform similarity. The MCC is then derived by selecting the lowest correlation value across all templates at each time point, implementing a conservative worst-case template-agreement criterion. In the feature extraction and prediction phase, statistical features such as skewness and the RoA are computed and used as inputs for a quadratic discriminant classifier to classify candidate windows as valid or invalid. Following classification, peak translation and constraint analysis refine the results by enforcing physiological plausibility constraints, such as heart rate limits and waveform symmetry, while aligning detected peaks with the original signal. This structured approach supports accurate and reliable peak detection under the tested clean and synthetic pulse-free-noise conditions.

## 4. Results

PCPD was evaluated using two distinct data sources: a 50-subject proposed dataset and 53 BIDMC recordings. In the clean evaluation, 86,661 manually verified real peaks were assessed. In the noisy evaluation, 134,643 local-maximum candidates were analyzed, encompassing both true pulse candidates and non-pulse noise candidates. The detailed performance results provide a comparative analysis of the effectiveness of the method. To assess the ability of PCPD to detect true pulse peaks under clean signal conditions, a first analysis was conducted on signals without added noise. The results of this evaluation are summarized in Table 2. Because no true negatives exist in the clean condition, the reported summary score is the mean of precision and recall rather than standard accuracy. The method achieved a balanced precision–recall score of 99.13% on the proposed dataset when compared to a manually verified ground truth of pulse peaks. Similarly, on the BIDMC dataset, the method demonstrated a balanced precision–recall score of 97.04%, indicating a strong ability to identify pulse peaks in the absence of added noise.

The primary objective of this work was to develop a peak detection algorithm capable of identifying true pulse peaks while minimizing false detections due to synthetic pulse-free noise. The results are presented in Table 3, which provides a comparative analysis of the effectiveness of the method. These results indicate that the method can distinguish valid pulse peaks from non-informative maxima under the tested controlled-noise protocol.

**Figure 9:**
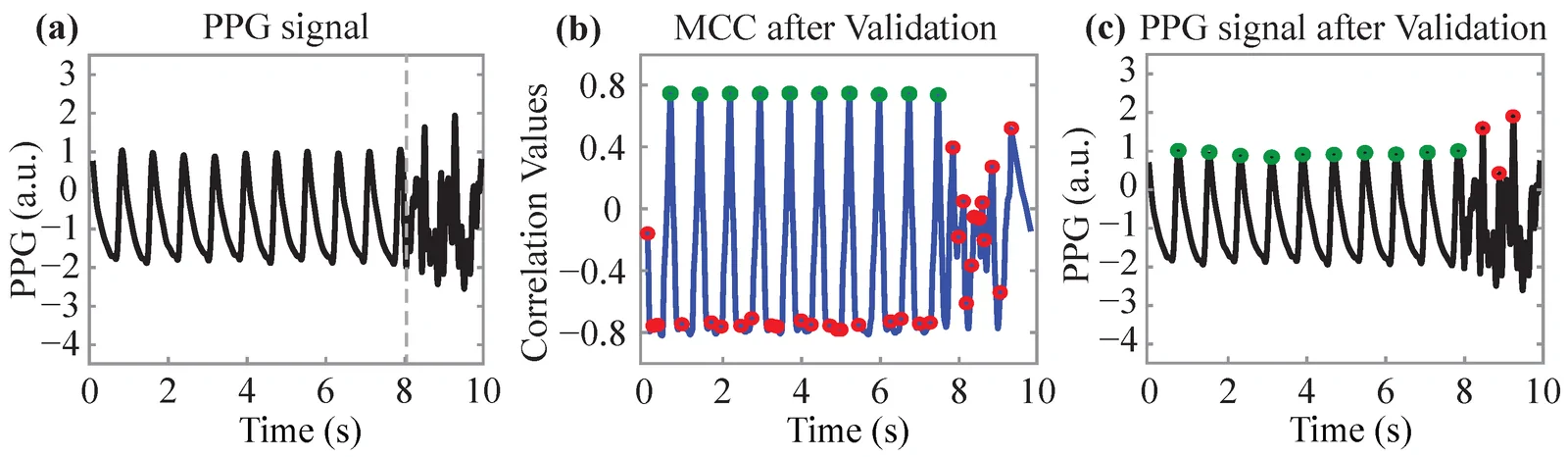
Results of peak detection using PCPD. (**a**) Example of a BIDMC PPG signal after band-pass filtering (0.5–8 Hz; Section 3.6). The dashed line separates the clean part of the signal on the left from the manually added synthetic noisy segment on the right. (**b**) MCC after validation: red peaks are classified as invalid, while green peaks are valid. (**c**) The initial PPG signal with validated (green) and invalidated (red) peaks. In this example, peaks in the clean segment are correctly identified, while peaks in the noisy section are not validated as true peaks.

As depicted in Figure 9, the method differentiates between the characteristic morphology of a valid pulse peak and synthetic non-pulse noise. The ability of the method to maintain high classification performance across the two evaluated datasets supports its promise as a PPG-only peak detector. The achieved accuracy, precision, recall, and F1 score indicate that the method rejects the injected pulse-free noise under the tested synthetic-noise conditions. Because accuracy depends on the class balance of the candidate set, the balanced accuracy (mean of recall and specificity) is also reported: 99.27% on the proposed dataset and 97.17% on BIDMC.

## 5. Discussion

This section discusses the findings obtained from the evaluation of PCPD. It presents an overview of the primary results, including a comparison of the performance of the method across the two datasets described above, and compares these results with those obtained using eleven existing benchmark methods for peak detection evaluated on the same datasets. This comparison allows the strengths and limitations of PCPD to be assessed relative to established techniques in the field.

### 5.1. Overview of Findings

PCPD demonstrated strong performance across both clean and synthetic-noise datasets, showing its ability to distinguish true pulse peaks from controlled non-pulse noise candidates.

On clean signals, the method exhibited strong results, with balanced precision–recall scores of 99.1% on the proposed dataset (internal performance) and 97.0% on the independent BIDMC dataset. This highlights that the method is effective in ideal, clean conditions. When applied to the noisy datasets, the method achieved high accuracy, precision, recall, and F1 score, with results indicating an ability to identify valid pulse candidates while minimizing false detections under synthetic pulse-free noise. Specifically, the method delivered an accuracy of 99.3% on the proposed dataset with added noise and 97.1% on the BIDMC dataset with noise. Key differences between the two datasets were nevertheless evident in the performance metrics. Under noise, both precision and recall were lower on the BIDMC dataset than on the proposed dataset (96.82% and 97.31% versus 99.30% and 99.03%, respectively). This difference is driven by a larger number of false positives on BIDMC (852 versus 232), which reduces precision, together with a larger number of missed pulse candidates (719 versus 320), which reduces recall, whereas the proposed dataset maintained more consistent results across the metrics. This gap is consistent with a degree of domain dependence: both the 16 ideal-beat templates and the binary classifier are built exclusively from the proposed dataset (Section 3.2 and Section 3.4), so some advantage on that dataset relative to an unseen, independently collected dataset such as BIDMC is expected, in addition to any dataset-specific differences in noise characteristics or signal morphology. Because performance on subjects of the proposed dataset not used to build the templates or to train the classifier is not lower (Section 5.4), these results support domain dependence (device, population, signal morphology) as the main explanation of the gap to BIDMC, although they do not exclude a contribution of subject overlap.

### 5.2. Impact of Noise on Beat Detection

**Figure 10:**
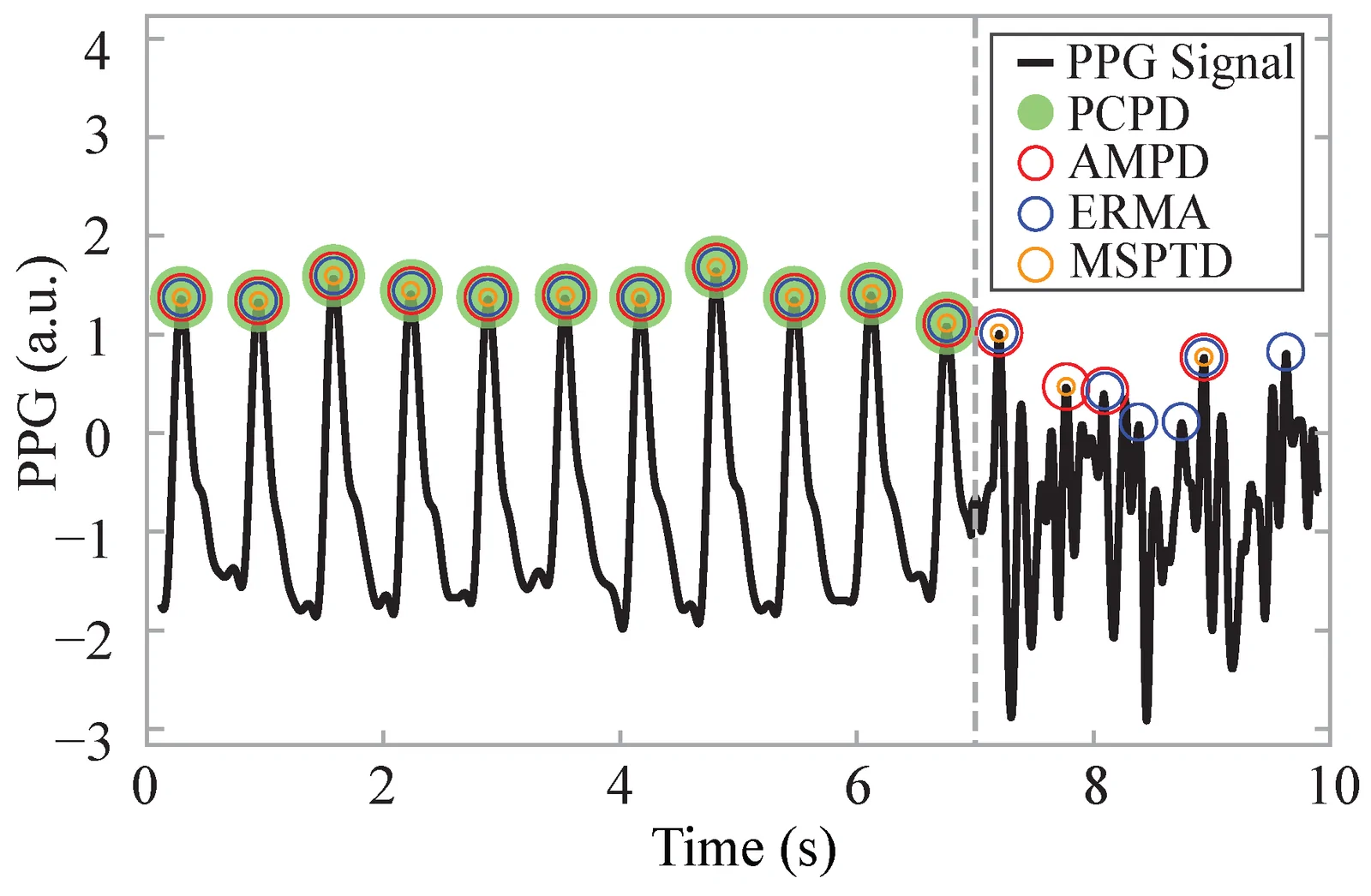
Performance of beat detectors compared in a segment with noise (located to the right of the dashed line). The three beat detectors compared with PCPD were selected as they achieved the best performance on the entire dataset. It can be observed that in the clean part of the signal, the beat detectors perform well, but their accuracy drops markedly when noise is introduced.

The addition of synthetic pulse-free noise presented a considerable challenge in the peak detection task, yet the proposed method demonstrated resilience against such controlled disturbances. The noise introduced into the signal simulates non-informative intervals such as temporary sensor detachment, but it does not cover the full variability of real motion artifacts. As illustrated in Figure 10, PCPD, compared with the other methods evaluated, differentiates between valid pulse peaks and synthetic pulse-free noise, reducing the likelihood that noise-induced peaks are misclassified as true signals. This capability is particularly critical in signal processing applications where precision is essential to prevent misinterpretation of data.

The effectiveness of the method in handling the tested synthetic noise can be attributed to the design of the MCC and its features, which enable it to maintain high performance in the presence of non-informative segments. When compared to other evaluated methods, which tend to degrade under this protocol, PCPD maintained high detection accuracy under this protocol.

The method also shows potential for future signal quality assessment (SQA). Research has highlighted the challenges in reliably distinguishing high-quality signal segments from noisy ones for accurate pulse wave analysis [31,32,33]. PCPD could contribute to these workflows by identifying segments dominated by invalid candidate peaks and filtering low-quality intervals early in the analysis. However, this manuscript evaluates PCPD primarily as a peak detector; a dedicated segment-level SQA validation against quality labels is still required before presenting it as a complete SQA method.

### 5.3. Comparison with Benchmark Methods in Beat Detection

Based on existing literature, eleven benchmark beat detection methods were selected for comparison. These methods incorporate various preprocessing steps and detection techniques, summarized in Table 4. The comparison should be interpreted as an evaluation of the selected implementations and parameter settings under the same clean and synthetic-noise protocols used for PCPD. The eleven benchmark methods were evaluated using their original, default parameter settings as provided in the ppg-beats toolbox [14] (version 1.0), without any additional tuning; the toolbox’s detector functions accept only the input signal and its sampling frequency, with no exposed parameters to adjust, so this default configuration is the only one available for each method. The ideal-beat templates and the binary classifier were developed on segments of the proposed dataset, and the evaluation on that dataset includes all 4462 Grade-4 segments. Development and evaluation segments were therefore not kept separate, and the results on the proposed dataset should be interpreted as internal performance; subject-independent estimates are given in Section 5.4. The BIDMC dataset was not used at any stage of development and provides a cross-dataset evaluation.

**Figure 11:**
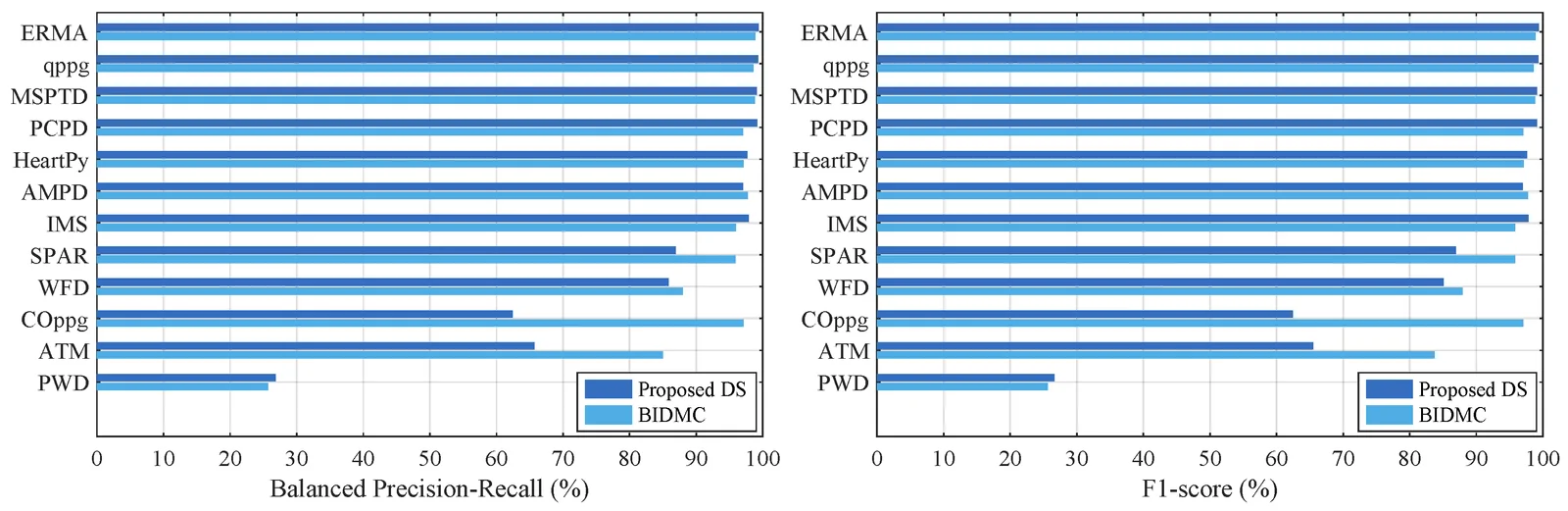
Comparative analysis of the eleven benchmark beat detection methods and the proposed PCPD method. The left panel illustrates the balanced precision–recall score across two different clean datasets, while the right panel presents the corresponding F1 scores, providing a complementary evaluation of model effectiveness.

**Figure 12:**
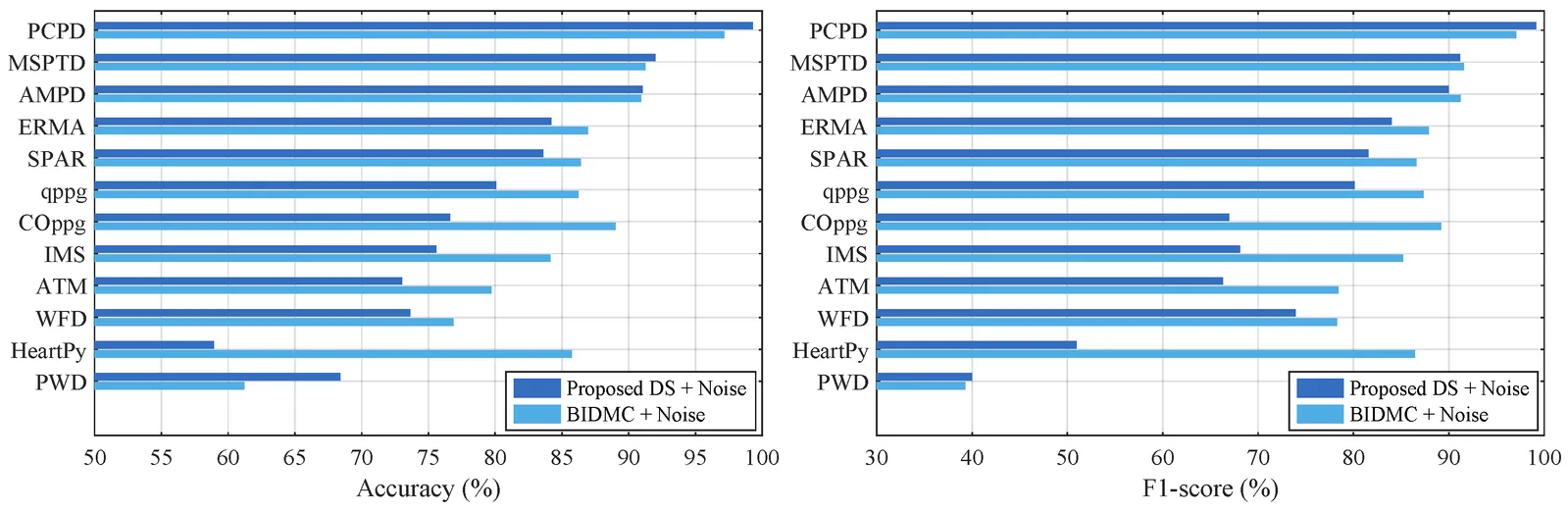
Comparative analysis of the eleven benchmark beat detection methods and the proposed PCPD method. The left panel illustrates accuracy across two different datasets with added synthetic pulse-free noise, while the right panel presents the corresponding F1 scores, providing a complementary evaluation of model effectiveness.

Table 5, Table 6, Table 7 and Table 8 report the complete confusion-matrix-derived metrics (TPs, FPs, FNs, TNs where applicable, precision, recall/sensitivity, specificity where applicable, and F1-score) underlying the accuracy and F1-score summaries shown in Figure 11 and Figure 12. The clean benchmark comparison (Table 5), the clean ablation study and the sensitivity analysis (Section 5.6) use the same unfiltered signal and the same reference set as Table 2 (49,626 peaks); the PCPD row of Table 5 and the full-model row of the clean ablation study therefore coincide with Table 2. The clean benchmark comparison uses this unfiltered signal so that all methods receive identical input and are scored against the same reference peaks, as in the noisy condition; each benchmark applies its own internal preprocessing (Table 4), so an additional external filter would duplicate it. The conference paper [19] had used a band-pass-filtered (0.5–8 Hz) version of the signal for the benchmark methods; results on that version are given in Appendix A (Table A1). All detectors were applied to the native 55-Hz signal, with the sampling frequency passed to each function and without resampling, as provided by the toolbox, which was itself assessed on 64-Hz wrist PPG [14]. To characterize the variability of PCPD’s performance across the evaluation set, per-segment accuracy was computed for each of the 4462 noisy segments in the proposed dataset and the 2544 noisy segments in BIDMC. PCPD achieved a mean per-segment accuracy of 99.32% (SD = 2.17%, 95% CI [99.26%, 99.39%]) on the proposed dataset and 97.08% (SD = 5.06%, 95% CI [96.89%, 97.28%]) on BIDMC, describing limited variability across the analyzed segments. As an exploratory segment-level comparison with the two strongest benchmark methods, the same per-segment accuracy was computed for MSPTD and AMPD on the same segments and compared with PCPD’s using a paired bootstrap (20,000 resamples) and a one-sided Wilcoxon signed-rank test (Table 9); both tests yielded p<0.001 in the two datasets. Because multiple segments can originate from the same subject or recording, these analyses do not account for within-subject clustering and should not be interpreted as subject-level confirmatory inference (see Section 5.4 for a subject-level analysis).

The comparative evaluation of eleven beat detection methods, plus the proposed PCPD method, was conducted across two clean datasets: the proposed dataset and BIDMC. As shown in Figure 11, most methods achieve high balanced precision–recall scores and F1 scores in these high-quality conditions, consistently identifying the correct peaks throughout the datasets, the main exceptions being PWD and ATM on both datasets and COppg on the proposed dataset. Because detected peaks are matched to reference peaks at the exact sample index, detectors whose output is displaced from the reference maximum are penalized; on the proposed dataset, for instance, ATM matches 62.0% of the reference peaks exactly and 84.1% within one sample. Similarly, PWD locates peaks from zero crossings of the first derivative, so its output is often displaced by one to three samples: 24.5% of the reference peaks are matched exactly and 77.0% within three samples. In addition to the clean data, the same methods were tested under noisy conditions by introducing synthetic pulse-free noise into the datasets. As illustrated in Figure 12, PCPD achieved the best performance among the evaluated implementations, consistently achieving higher accuracy and F1 score under the tested protocol. This reflects the ability of PCPD to identify beat morphology in the presence of controlled signal perturbations. The results suggest that the sensitivity of PCPD to morphological structures is a key factor contributing to its resilience under noisy conditions. Among the benchmark methods, MSPTD [15] and AMPD [17] show the strongest performance aside from PCPD. These approaches utilize scalogram-based multi-scale peak detection, enabling them to adapt to morphological variations and to increase their robustness to noise. ERMA [35], which relies on moving averages to smooth and emphasize peak structures, also performs relatively well, underscoring the value of preprocessing strategies that simplify peak morphology.

These findings underscore the importance of methods that effectively capture and preserve the fundamental shape of PPG waveforms while mitigating the impact of non-informative intervals. In contrast, approaches that depend primarily on thresholding or simple derivative-based criteria tend to suffer greater performance degradation under the tested noisy conditions. The advantage of PCPD is specific to the synthetic-noise condition. On clean signals, PCPD performs comparably to, but not better than, the strongest benchmarks: ERMA and qppg obtain higher F1-scores on the proposed dataset (Table 5), and AMPD, ERMA, MSPTD and qppg on BIDMC (Table 6). On clean BIDMC, most of the 1524 false positives of PCPD (about 94%) fall between 20% and 60% of the inter-beat interval after a reference peak, i.e., on the diastolic wave, and about two thirds come from five of the 53 recordings. This is consistent with templates and classifier derived from wrist reflectance PPG, whose diastolic features are weak, applied to finger transmission PPG with a pronounced dicrotic wave. Even under synthetic noise, the advantage cannot be extrapolated to real motion artifacts, which typically retain quasi-periodic components that may be confused with genuine pulses.

Beyond the classical benchmark methods evaluated above, deep learning has also been explored for PPG-based cardiac signal analysis, notably convolutional neural networks trained end-to-end on time-frequency representations to estimate heart rate under motion artifacts [18]. These approaches offer a complementary ability to learn complex signal representations, whereas PCPD relies on two explicit features—skewness and RoA—that make each candidate-level decision traceable to defined geometric properties. A direct comparison with a deep-learning detector was not performed; therefore, no conclusion can be drawn here regarding relative accuracy, computational requirements, or training-data requirements. Such a comparison is left for future work.

Relative to the conference paper [19], the following figures and descriptions have been corrected: the F1-score on clean BIDMC signals is 97.03% (reported there as 96.92%; precision and recall are unchanged); the noisy evaluation comprises 134,643 candidates, 44.44% valid and 55.56% invalid (reported there as 139,953, 47%/53%, an erroneous total; the PCPD results were computed on the same candidates); the clean benchmark comparison uses the same unfiltered signal as PCPD, whereas the conference paper used a band-pass-filtered version for the benchmark methods, which changes some benchmark scores (Table 5); the peak-translation window is ±10 samples (±182 ms at 55 Hz, ±80 ms at 125 Hz), described there as ±90 ms; and the classifier training data and validation are those of Section 3.4 (2002 segments, about 5.6 h, single segment-level hold-out), rather than the seven hours and five-fold cross-validation reported there.

### 5.4. Subject-Independent Evaluation

To obtain estimates free of development overlap, PCPD was evaluated in two subject-independent ways. (i) Held-out subjects: the original model was applied to the 29 subjects not used to build the templates or to train the classifier (subjects 22–50), and the benchmark methods were scored on the same segments. (ii) Subject-wise five-fold cross-validation: the 50 subjects were split into five folds of ten, stratified by number of Grade-4 segments. In each fold the 16 templates and the classifier were rebuilt from the training subjects only, with the same procedures and pool size, and PCPD was evaluated on the held-out subjects with unchanged thresholds. Because the original feature-extraction script was not preserved, the classifier was re-implemented for this analysis; trained on the original pool, the re-implementation changes F1 by up to 0.7 points relative to Table 2. Results are summarized in Table 10.

Performance on held-out subjects did not decrease (F1 99.50% clean; accuracy 99.50% noisy). With templates and classifier rebuilt in each fold, F1 was 98.93% on clean signals and accuracy 98.87% under synthetic noise; per subject, noisy accuracy had a median of 99.04% (range 92.37–100%). Under noise, PCPD exceeded MSPTD and AMPD in all 50 subjects (median difference +7.1 and +7.9 percentage points; two-sided Wilcoxon signed-rank test with the subject as unit, p<0.001). The lowest fold (noisy accuracy 96.90%) is the only one whose templates came from a different subject, indicating some dependence on the template source. On clean signals, PCPD remained comparable to, but not better than, the strongest benchmarks (e.g., ERMA 99.66% and qppg 99.64% F1 on held-out subjects).

### 5.5. Study Limitations

While PCPD demonstrated strong performance under the tested clean and synthetic-noise conditions, several limitations should be acknowledged. The proposed dataset includes subjects with a wide range of body mass index, with 60% classified as overweight or obese; however, the performance of PCPD was not separately analyzed as a function of BMI, and it remains unknown whether detection accuracy is affected by the altered PPG waveform morphology and reduced signal amplitude often associated with higher adiposity. Similarly, the method has not been specifically evaluated under conditions of peripheral hypoperfusion, where weak pulse waves and low signal-to-noise ratio can substantially degrade PPG quality. The self-collected dataset additionally contains real, non-synthetic signal degradation in the form of Grade 2 and Grade 3 segments (26.6% of the dataset, more than 2300 ten-second segments), which were not included in the quantitative evaluation. These grades were assigned at the segment level, based on the proportion of the window with recognizable pulses, and do not specify which individual local maxima within a segment correspond to genuine, distorted pulses as opposed to motion-artifact maxima; because real artifacts often retain quasi-periodic components that can resemble true pulses, extending the candidate-level TP/FP/TN/FN framework used in this study to this subset would require a dedicated peak-level annotation of these segments, which was not performed here. As noted in Section 2.2, this protocol allows assessment of the ability of the method to detect true peaks while rejecting noise-induced maxima, but it should not be interpreted as a full validation under real motion artifacts. In addition, the classifier split was performed at the segment level rather than the subject level, the segment-level comparisons of Table 9 do not account for within-subject clustering (a subject-level test is reported in Section 5.4), and the segments used to develop the templates and the classifier were not excluded from the evaluation on the proposed dataset, so those results represent internal performance; held-out-subject and subject-wise cross-validated results are reported in Section 5.4. The templates are derived from a single subject, and cross-validation showed some dependence on this choice. The benchmark methods were evaluated using the default implementations available in the selected toolbox, whereas PCPD was developed on the proposed data; consequently, the comparative results apply to these implementations and settings rather than establishing universal superiority over each benchmark method. Future work should therefore include stratified analyses across BMI categories and perfusion states, a peak-level annotation and evaluation of the real Grade 2/3 segments, validation on datasets containing naturally occurring PPG artifacts, and validation on independent subject cohorts and acquisition devices to assess generalization beyond the present dataset and hardware.

### 5.6. Computational Cost and Parameter Sensitivity

On a desktop-class computer (Intel Core i7-8750H, single-threaded MATLAB R2023b), PCPD processes a 10-s segment in 512.9±13.8 ms (mean ± SD over 60 clean segments), a real-time factor of approximately 19.5×. The template-count analysis of Figure 4 was run on a different computer (approximately 360 ms per segment with 16 templates); absolute times therefore depend on hardware, while the linear dependence on the number of templates holds on both. The result demonstrates real-time execution only for the tested computer and software configuration. No direct runtime comparison was performed using the same hardware and implementation environment, and the experiment does not establish latency, memory use, energy consumption, or feasibility on embedded wearable hardware; these aspects require platform-specific implementation and evaluation.

To quantify the contribution of each post-classification constraint, the heart-rate-range constraint, the edge-symmetry constraint, and the binary classifier were each removed in turn and the method re-evaluated on the same full official clean and noisy datasets used in Section 2.2 (4462 segments in both conditions). Results are shown in Table 11 (noisy condition) and Table 12 (clean condition).

On noisy data, all three constraints contribute to performance, although the effect of removing the heart-rate-range constraint is modest, with the classifier as the dominant driver of noise robustness (removing it costs 3.59 F1 points and increases false positives 12-fold). On clean data, by contrast, the same constraints have a negligible or even slightly negative effect on F1, indicating that they act specifically as noise-rejection mechanisms rather than general-purpose filters: they cost essentially nothing when the signal is clean and intervene decisively only when needed.

The sensitivity of PCPD to its main thresholds was assessed by varying each parameter individually in an exploratory one-at-a-time analysis on the full clean proposed dataset (4462 segments; 49,626 reference peaks) and is summarized in Table 13. This analysis evaluates local robustness on this dataset and is not an independent joint optimization or external validation of the thresholds.

The edge-symmetry threshold is stable up to 0.5, loses 0.5 F1 points at 0.6 and degrades sharply at 0.7. Varying the upper heart-rate bound between 180 and 240 bpm has no measurable effect. Narrowing the peak-translation window below ±10 samples reduces F1 (98.96% at ±7 samples and 97.25% at ±5 samples), whereas widening it to ±15 samples yields no meaningful gain. Repeating the same one-at-a-time analysis on the full noisy proposed dataset, where the constraints act as noise-rejection mechanisms, gave the same picture: the edge-symmetry threshold of 0.5 yields the highest F1 (99.17%, compared with 98.93–99.08% at 0.3–0.4, 98.81% at 0.6 and 83.38% at 0.7); the peak-translation window reaches its plateau at ±10 samples (99.14% at ±7 and 99.15% at ±15 samples, but 98.33% at ±5 and 83.36% at ±3 samples); and varying the upper heart-rate bound between 180 and 240 bpm changes F1 by less than 0.1 percentage points (99.11–99.20%), lower bounds rejecting slightly more noise candidates. These results support local stability of the selected values on this dataset but do not establish global optimality or generalization to other datasets.

## 6. Conclusions

PCPD is a novel method for detecting heartbeats in PPG signals under clean and controlled synthetic-noise conditions. The algorithm operates through a five-step pipeline: (1) signal segmentation with overlap to ensure comprehensive peak capture, (2) sliding cross-correlation with high-quality template beats to assess waveform similarity, (3) derivation of the MCC to emphasize the least favorable alignment across templates, (4) machine learning-based classification using features such as skewness and the RoA, and (5) peak translation and constraint analysis.

PCPD was evaluated on a newly created 50-subject dataset with manually labeled signal quality grades and on 53 recordings from the publicly available BIDMC dataset. In the clean condition, 86,661 manually verified real peaks were evaluated; in the noisy condition, 134,643 local-maximum candidates were analyzed, encompassing both true pulse candidates and non-pulse noise candidates. On clean signals, PCPD achieved balanced precision–recall scores of 99.1% on the proposed dataset (internal performance) and 97.0% on the independent BIDMC dataset. In synthetic pulse-free-noise conditions, PCPD maintained high standard accuracy, with 99.3% on the proposed dataset and 97.1% on BIDMC. These results highlight the ability of the method to distinguish valid pulse peaks from synthetic noise-induced artifacts under the tested protocol. One of the key practical advantages of PCPD is its PPG-only design, which enables peak detection without requiring auxiliary signals such as ECG during deployment; nonetheless, an independent physiological reference such as ECG would further strengthen future validation of beat detection performance. Additionally, PCPD may be useful for future SQA workflows if segment-level quality validation is added.

In conclusion, PCPD represents a promising approach for PPG-based heartbeat detection, offering a noise-resilient and accurate solution under the evaluated clean and synthetic pulse-free-noise conditions. Its ability to operate independently of auxiliary signals suggests potential for broader use in wearable PPG monitoring, although validation under real motion artifacts remains necessary. 

## Figures and Tables

**Table 1:** Distribution of Signal Quality Grades. The table presents the percentage distribution of PPG signal segments classified into four quality grades based on the recognizability of PPG pulse morphology. Grade 1 represents segments with no reliably recognizable PPG pulse morphology, while Grade 4 includes segments with consistently recognizable PPG pulse morphology throughout the entire segment.

Quality Grade	Description	Percentage
1	No recognizable PPG pulses	21.75%
2	<50% recognizable PPG pulses	12.26%
3	>50% recognizable PPG pulses	14.35%
4	Consistently recognizable PPG pulses	51.64%

**Table 2:** Performance comparison of PCPD on clean signals from the proposed dataset and BIDMC dataset. The Balanced Score is the mean of precision and recall because true negatives are not defined for clean peak-only evaluation. All performance metrics are expressed as percentages.

Dataset	Real Peaks	Detected Peaks	Balanced Score	Precision	Recall	F1 Score
Proposed dataset	49,626	48,866	99.13	99.90	98.37	99.12
BIDMC	37,035	37,860	97.04	95.97	98.11	97.03

**Table 3:** Performance comparison of PCPD on the two different datasets with the addition of manual noise in each segment. All performance metrics are expressed as percentages.

Dataset	TPs	FNs	FPs	TNs	Accuracy	Precision	Recall	F1 Score
Proposed Dataset + Noise	32,836	320	232	45,922	99.30	99.30	99.03	99.17
BIDMC + Noise	25,962	719	852	27,800	97.16	96.82	97.31	97.06

**Table 4:** The eleven PPG beat detection methods used for comparison with PCPD. Each method is described by its preprocessing and its detection strategy. LMS: local maxima scalogram.

Method	Preprocessing	Description
AMPD [17]	Detrend, 6 s windows	Local maxima scalogram (LMS) for multi-scale peak detection
ATM [34]	Bandpass 0.5–20 Hz	Local minima below an adaptive threshold
COppg [23]	10 s window, adaptive filter	Peaks above the 90th percentile
ERMA [35]	Bandpass 0.5–8 Hz, rectification	Moving averages to emphasize peaks and beats
HeartPy [36]	Normalization, squaring	Maxima above a moving average
IMS [37]	Continuous positive gradients	Adaptive thresholds for amplitude and duration
MSPTD [15]	LMS for peaks and minima	AMPD modification with computational improvements
PWD [38]	First derivative of PPG	Zero-crossing for onset and peak identification
qppg [39]	Gradient sum function	Detection of systolic upslopes
SPAR [40]	7D phase space representation	Attractor reconstruction to detect beats
WFD [41]	Bandpass 0.5–8 Hz, wavelet	Zero-crossing of third derivative to detect beats

**Table 5:** Confusion-matrix-derived detection performance of PCPD and the eleven benchmark methods on the clean proposed dataset (OUR DS; 49,626 reference peaks). TPs: true positives; FPs: false positives; FNs: false negatives.

Method	TPs	FPs	FNs	Precision (%)	Recall (%)	F1 (%)
PCPD	48,815	51	811	99.90	98.37	99.12
AMPD	47,947	1300	1679	97.36	96.62	96.99
ATM	30,773	13,560	18,853	69.41	62.01	65.50
COppg	30,449	17,498	19,177	63.51	61.36	62.41
ERMA	49,137	120	489	99.76	99.01	99.38
HeartPy	47,365	86	2261	99.82	95.44	97.58
IMS	47,512	18	2114	99.96	95.74	97.81
MSPTD	48,946	198	680	99.60	98.63	99.11
PWD	12,164	29,560	37,462	29.15	24.51	26.63
qppg	49,177	239	449	99.52	99.10	99.31
SPAR	43,399	6821	6227	86.42	87.45	86.93
WFD	46,684	13,438	2942	77.65	94.07	85.07

**Table 6:** Confusion-matrix-derived detection performance of PCPD and the eleven benchmark methods on the clean BIDMC dataset. TPs: true positives; FPs: false positives; FNs: false negatives.

Method	TPs	FPs	FNs	Precision (%)	Recall (%)	F1 (%)
PCPD	36,336	1524	699	95.97	98.11	97.03
AMPD	36,375	1008	660	97.30	98.22	97.76
ATM	27,619	1337	9416	95.38	74.58	83.71
COppg	35,271	397	1764	98.89	95.24	97.03
ERMA	36,420	192	615	99.48	98.34	98.90
HeartPy	35,347	430	1688	98.80	95.44	97.09
IMS	34,198	131	2837	99.62	92.34	95.84
MSPTD	36,721	585	314	98.43	99.15	98.79
PWD	9176	25,352	27,859	26.58	24.78	25.64
qppg	36,629	629	406	98.31	98.90	98.61
SPAR	36,440	2568	595	93.42	98.39	95.84
WFD	33,140	5195	3895	86.45	89.48	87.94

**Table 7:** Confusion-matrix-derived detection performance of PCPD and the eleven benchmark methods on the noisy proposed dataset (OUR DS). TPs: true positives; FPs: false positives; FNs: false negatives; TNs: true negatives.

Method	TPs	FPs	FNs	TNs	Precision (%)	Recall (%)	Specificity (%)	F1 (%)
PCPD	32,836	232	320	45,922	99.30	99.03	99.50	99.17
AMPD	32,011	5970	1145	40,184	84.28	96.55	87.07	90.00
ATM	21,072	9311	12,084	36,843	69.35	63.55	79.83	66.33
COppg	18,808	4203	14,348	41,951	81.73	56.73	90.89	66.97
ERMA	32,901	12,267	255	33,887	72.84	99.23	73.42	84.01
HeartPy	16,930	16,336	16,226	29,818	50.89	51.06	64.61	50.98
IMS	20,679	6887	12,477	39,267	75.02	62.37	85.08	68.11
MSPTD	32,802	5995	354	40,159	84.55	98.93	87.01	91.18
PWD	8356	273	24,800	45,881	96.84	25.20	99.41	40.00
qppg	31,807	14,444	1349	31,710	68.77	95.93	68.70	80.11
SPAR	28,746	8594	4410	37,560	76.98	86.70	81.38	81.55
WFD	29,619	17,365	3537	28,789	63.04	89.33	62.38	73.92

**Table 8:** Confusion-matrix-derived detection performance of PCPD and the eleven benchmark methods on the noisy BIDMC dataset. TPs: true positives; FPs: false positives; FNs: false negatives; TNs: true negatives.

Method	TPs	FPs	FNs	TNs	Precision (%)	Recall (%)	Specificity (%)	F1 (%)
PCPD	25,962	852	719	27,800	96.82	97.31	97.03	97.06
AMPD	26,012	4343	669	24,309	85.69	97.49	84.84	91.21
ATM	20,411	4949	6270	23,703	80.49	76.50	82.73	78.44
COppg	25,094	4490	1587	24,162	84.82	94.05	84.33	89.20
ERMA	26,186	6733	495	21,919	79.55	98.14	76.50	87.87
HeartPy	25,116	6327	1565	22,325	79.88	94.13	77.92	86.42
IMS	25,194	7284	1487	21,368	77.57	94.43	74.58	85.17
MSPTD	26,229	4379	452	24,273	85.69	98.31	84.72	91.57
PWD	6943	1729	19,738	26,923	80.06	26.02	93.97	39.28
qppg	26,234	7175	447	21,477	78.52	98.32	74.96	87.32
SPAR	24,335	5179	2346	23,473	82.45	91.21	81.92	86.61
WFD	23,024	9131	3657	19,521	71.60	86.29	68.13	78.27

**Table 9:** Exploratory segment-level paired comparison of PCPD’s per-segment accuracy against the two strongest benchmark methods (MSPTD, AMPD), on the same segments underlying Table 7 and Table 8. Mean Δ: mean per-segment accuracy difference (PCPD minus benchmark), in percentage points; CI from a paired bootstrap (20,000 resamples); *p*-value from a one-sided Wilcoxon signed-rank test.

Dataset	Comparison	Mean Δ (pp)	95% CI (pp)	*p*-Value
Proposed dataset	PCPD vs. MSPTD	+6.69	[6.56, 6.82]	<0.001
Proposed dataset	PCPD vs. AMPD	+7.73	[7.58, 7.88]	<0.001
BIDMC	PCPD vs. MSPTD	+5.24	[5.00, 5.48]	<0.001
BIDMC	PCPD vs. AMPD	+5.59	[5.35, 5.83]	<0.001

**Table 10:** Subject-independent evaluation on the proposed dataset. Held-out: subjects 22–50, never used for template or classifier development; benchmark methods are scored on the same segments. Cross-validation (CV): subject-wise five-fold, with templates and classifier rebuilt in each fold. P: precision; R: recall; Acc: accuracy; Spec: specificity. All values in %.

			Clean			Noisy		
Setting	Subjects	Segments	P	R	F1	Acc	F1	Spec
PCPD, all subjects (Table 2 and Table 3)	50	4462	99.90	98.37	99.12	99.30	99.17	99.50
PCPD, held-out	29	3094	99.94	99.05	99.50	99.50	99.42	99.49
MSPTD, held-out	29	3094	99.71	99.29	99.50	91.94	91.41	86.30
AMPD, held-out	29	3094	97.52	97.33	97.43	90.98	90.27	86.36
PCPD, subject-wise CV	50	4462	99.80	98.08	98.93	98.87	98.65	98.96

**Table 11:** Ablation study on the noisy proposed dataset (4462 segments; 33,156 clean-region reference peaks; 46,154 noise-region candidates). TPs: true positives; FPs: false positives; FNs: false negatives; TNs: true negatives.

Variant	TPs	FPs	FNs	TNs	Precision (%)	Recall (%)	Specificity (%)	F1 (%)
Full model (PCPD)	32,836	232	320	45,922	99.30	99.03	99.50	99.17
Without HR-range constraint	32,837	390	319	45,764	98.83	99.04	99.16	98.93
Without symmetry constraint	32,840	742	316	45,412	97.79	99.05	98.39	98.41
Without classifier	32,908	2799	248	43,355	92.16	99.25	93.94	95.58

**Table 12:** Ablation study on the clean proposed dataset (4462 segments; 49,626 reference peaks). TN is not defined for clean peak-only evaluation. TPs: true positives; FPs: false positives; FNs: false negatives.

Variant	TPs	FPs	FNs	Precision (%)	Recall (%)	F1 (%)
Full model (PCPD)	48,815	51	811	99.90	98.37	99.12
Without HR-range constraint	48,817	60	809	99.88	98.37	99.12
Without symmetry constraint	48,850	85	776	99.83	98.44	99.13
Without classifier	49,095	138	531	99.72	98.93	99.32

**Table 13:** Sensitivity of PCPD to its main thresholds, evaluated on the full clean proposed dataset (4462 segments; 49,626 reference peaks). The value used in the main pipeline is marked in bold.

Parameter	Tested Value	F1 (%)
Edge-symmetry threshold	0.3	99.13
	0.4	99.12
	**0.5 (used)**	**99.12**
	0.6	98.64
	0.7	82.86
Heart-rate range (upper bound)	180 bpm	99.12
	**220 bpm (used)**	**99.12**
	240 bpm	99.12
Peak-translation window	±3 samples (±55 ms)	72.06
	±5 samples (±91 ms)	97.25
	±7 samples (±127 ms)	98.96
	**±10 samples (±182 ms) (used)**	**99.12**
	±15 samples (±273 ms)	99.14

## Data Availability

The data supporting reported results and presented in this study are available on request from the corresponding author.

## Appendix A. Clean Benchmark Comparison on the Filtered Signal

Table A1 reports the clean benchmark comparison on the proposed dataset when the signal is band-pass filtered (fourth-order zero-phase Butterworth, 0.5–8 Hz) before detection, as in the benchmark comparison of the conference paper [19]. The reference peaks are obtained with the same procedure as in the main text, applied to the filtered signal (49,379 reference peaks, compared with 49,626 on the unfiltered signal of Table 5), and detected peaks are matched at the exact sample index. On the filtered signal, PWD detects peaks displaced by one sample from the reference maxima, which exact-index matching counts as false positives and false negatives.

**Table A1:** Confusion-matrix-derived detection performance of PCPD and the eleven benchmark methods on the clean proposed dataset after band-pass filtering (0.5–8 Hz; 49,379 reference peaks). TPs: true positives; FPs: false positives; FNs: false negatives.

Method	TPs	FPs	FNs	Precision (%)	Recall (%)	F1 (%)
PCPD	48,856	53	523	99.89	98.94	99.41
AMPD	47,111	2252	2268	95.44	95.41	95.42
ATM	42,928	2201	6451	95.12	86.94	90.85
COppg	46,442	1612	2937	96.65	94.05	95.33
ERMA	49,178	93	201	99.81	99.59	99.70
HeartPy	47,584	55	1795	99.88	96.36	98.09
IMS	47,825	23	1554	99.95	96.85	98.38
MSPTD	49,075	97	304	99.80	99.38	99.59
PWD	737	47,562	48,642	1.53	1.49	1.51
qppg	49,233	202	146	99.59	99.70	99.65
SPAR	43,577	6699	5802	86.68	88.25	87.46
WFD	48,727	11,397	652	81.04	98.68	89.00
